# Do More Hospital Beds Lead to Higher Hospitalization Rates? A Spatial Examination of Roemer’s Law

**DOI:** 10.1371/journal.pone.0054900

**Published:** 2013-02-13

**Authors:** Paul L. Delamater, Joseph P. Messina, Sue C. Grady, Vince WinklerPrins, Ashton M. Shortridge

**Affiliations:** 1 Department of Geography, Michigan State University, East Lansing, Michigan, United States of America; 2 Department of Geography, Center for Global Change and Earth Observations, Michigan AgBioResearch, Michigan State University, East Lansing, Michigan, United States of America; 3 Georgetown University Medical Center, Washington, D.C., United States of America; The University of Hong Kong, Hong Kong

## Abstract

**Background:**

Roemer’s Law, a widely cited principle in health care policy, states that hospital beds that are built tend to be used. This simple but powerful expression has been invoked to justify Certificate of Need regulation of hospital beds in an effort to contain health care costs. Despite its influence, a surprisingly small body of empirical evidence supports its content. Furthermore, known geographic factors influencing health services use and the spatial structure of the relationship between hospital bed availability and hospitalization rates have not been sufficiently explored in past examinations of Roemer’s Law. We pose the question, “Accounting for space in health care access and use, is there an observable association between the availability of hospital beds and hospital utilization?”

**Methods:**

We employ an ecological research design based upon the Anderson behavioral model of health care utilization. This conceptual model is implemented in an explicitly spatial context. The effect of hospital bed availability on the utilization of hospital services is evaluated, accounting for spatial structure and controlling for other known determinants of hospital utilization. The stability of this relationship is explored by testing across numerous geographic scales of analysis. The case study comprises an entire state system of hospitals and population, evaluating over one million inpatient admissions.

**Results:**

We find compelling evidence that a positive, statistically significant relationship exists between hospital bed availability and inpatient hospitalization rates. Additionally, the observed relationship is invariant with changes in the geographic scale of analysis.

**Conclusions:**

This study provides evidence for the effects of Roemer’s Law, thus suggesting that variations in hospitalization rates have origins in the availability of hospital beds. This relationship is found to be robust across geographic scales of analysis. These findings suggest continued regulation of hospital bed supply to assist in controlling hospital utilization is justified.

## Introduction

Roemer’s Law famously and simply states, *hospital beds that are built tend to be used* [1]. Although the authors’ original intent behind the statement is debatable, the most common interpretation is that as the supply of hospital beds increases the use of hospital services also increases. Roemer’s Law has fostered the belief that excess hospital beds leads to an *over*utilization of hospital services, when the observed demand outpaces the population’s actual need for services [2]. Hospital utilization rates rise, therefore, due to higher levels of inpatient admissions which may or may not lead to longer stays, contributing to higher costs. Wennberg [3] suggests that Roemer’s Law may be due to physicians being influenced by a subliminal knowledge regarding the availability of hospital beds.

In the USA, the high costs of inpatient hospitalizations, in conjunction with the generally accepted implications of Roemer’s Law, serve as the justification for state-based Certificate Of Need (CON) programs. CON programs are independent entities that are responsible for regulation of the supply of health care services such that the supply meets the population’s health care needs without an oversupply or duplication of services. Given that the plurality of overall health care expenditure in the USA is for inpatient hospital care [4], hospitalizations, and thus hospitals, are logical candidates for cost control measures. Supply is regulated by CON programs [5] wherein an unmet demand for services must be demonstrated prior to CON approval of new expenditures for hospital construction or expansion. Currently in the USA, 35 states have some form of CON program with 28 states specifically regulating the supply of acute care hospital beds [6].

Roemer’s Law defines a positive relationship between the availability of hospital beds and the use of hospital services. Past research has provided support for the effects of Roemer’s Law [3], [7]–[11], while other research has found conflicting [12]–[14] or inconclusive results [15]. The intertwined relationships among population health, access, use of health care services, and outcomes provide a number of research dilemmas, both theoretically and methodologically. Perhaps, the most difficult dilemma is defining and characterizing the availability of hospital beds. Although counting the number of beds in a hospital is trivial, measuring the overall availability of those beds *to a population* is a much more complex task and influenced by distance, demand, and access-related factors. Measures of hospital bed availability such as beds per county or minimum distance to a hospital [16], [17] ignore the multifaceted nature of access and the spatial and geographic nature of health care service use. Others have noted that the observed effects of Roemer’s Law may be due to oversimplified methods used to assign hospital beds to regions [18]. In addition, statistical methods that do not incorporate spatial structure in the relationship between access and utilization are at risk of being mis-estimated due to the effects of spatial autocorrelation.

As Wennberg and colleagues [19] have noted, *in American health care, geography is destiny*. The important role of spatial factors in health care services use have not been been given full consideration when exploring Roemer’s Law. Hence, we believe a substantive re-examination is warranted.

So, the critical question remains, “does the availability of hospital beds affect hospital utilization?”. Whereas Roemer’s natural experiment [20] was based on a regional study when a single hospital added a substantial number of inpatient beds, we approach this issue by examining an entire hospital system, comprising the hospitals, populations, and transportation infrastructure that connects populations to hospitals. We employ an ecological research design that integrates individual behavioral models of health care utilization in an explicitly spatial context. The research question is reframed to ask, “Accounting for space in health care access and use, is there an observable association between the availability of hospital beds and hospital utilization?”.

We characterize both the spatial and aspatial components of access such that their individual and combined contributions can be subsequently identified. Furthermore, by controlling for other determinants of hospital utilization, we isolate the effects of hospital bed availability on the utilization of hospital services, allowing us to statistically examine the effects of Roemer’s Law on hospitalization rates. In addition, we explore the stability of the relationship between hospital bed availability and hospital utilization by constructing models at varying scales of geographic analysis.

## Research Design

The Andersen model of health service utilization serves as the underlying theoretical framework in our research: utilization of health services results from a predisposing component, an enabling component, and illness level or “need” [21]. This framework is appealing because characteristics of both the population and the health care delivery system are integrated into a single model:
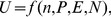
(1)where *U* is health services utilization, *n* is the number of people, *P* is the predisposing component, *E* is the enabling component, and *N* is need for services. The Anderson model has been recognized as most-commonly employed model for health service utilization studies [22].

The predisposing component in the Anderson model arises from the demographic structure of the population. We define:
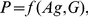
(2)where *Ag* and *G* are the age and gender structure of the population.

The enabling component in the Anderson model roughly equates to access, but does not provide the detailed characterization of access necessary to examine Roemer’s Law. Therefore, we extend the Andersen model using the theoretical framework offered by Penchansky and Thomas [23] that defines access as the “fit” between the population in need of services and services offered. In this framework, access results from a combination of five separate dimensions. Khan [24] classified the dimensions into spatial components: accessibility (*Ac*) and availability (*Av*) and aspatial components: affordability (*Af*), acceptability (*Ap*), and accommodation (*Am*). In addition to the five access components proposed, we add a mobility component (*M*) to capture differences in the ability to overcome distance [25]. We redefine the enabling component (*E*) as access (*A*) such that:
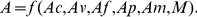
(3)


It is important to highlight the distinction between need (*N*) and demand (*U*) for services in the Anderson model. Although a certain amount of 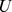 is predictable based on known demographic characteristics of the population, *N* arises from the general health status of the population [26] and, for hospitalizations, includes a stochastic element triggered by unpredictable instances of ill-health [27]. Measuring *N* is problematic in health services research given that patients and health professionals often evaluate the need for services differently [28], resulting in cases of both unmet need and unnecessary utilization. Oleske [29] report six approaches to measuring health care need, yet all are essentially proxies for estimating *H*. Therefore, we represent *N* as:
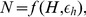
(4)where *H* describes the general health of the population and 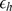 is a random variable representing occurrences of ill-health. Measuring *H* is problematic, as there is no measure that comprehensively characterizes the health status of populations. Therefore, we use socio-economic status (SES) measures, income (*In*), education (*Ed*), and ethnicity/race (*Et*) to capture variations in population health (see Young [30], pp.153–154 for a discussion of inclusion of ethnicity/race in health models):



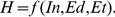
(5)Although there may be questions regarding causality between SES and health, SES has shown to be significantly correlated with both morbidity and/or self-assessed health status (see examples provided in [31]) in the US and internationally [30]. Given this relationship, SES-related variables act as potential predictors of variations in hospital utilization resulting from health-related conditions.

To control for potential confounding due to variations in *H* not fully captured in Eq. 5, we supplement our theoretical model by including Low Variation (LV) hospitalizations (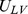). LV hospitalizations are those with little clinical-based doubt regarding the need for hospitalization [3]; from a clinical and/or epidemiological perspective, LV hospitalization rates can be considered as arising from the actual health care needs of the population [32], as they are generally unaffected by variations in hospital bed availability [19]. By adding LV hospitalization rate as an predictor variable in the model, we account for potential variability in hospital utilization that is justified due to the underlying needs of the population.

To further augment our theoretical model, we include hospitalizations for Ambulatory Care Sensitive (ACS) conditions (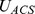) as an explanatory variable. These hospitalizations (also known as preventable hospitalizations) are considered avoidable if primary care is available [33] and accessible [34]. By including ACS hospitalizations in the model, we attempt to eliminate confounding due to variations in hospital utilization that result from inadequate access to primary care.

The theoretical model of hospital utilization is examined at an ecological level. Observation units include aggregated populations that reside in particular areal units or zones. Given that population sizes among areal units are dissimilar, we normalize all variables by population size, producing rate-based (e.g., beds/person) or proportional (e.g., % of population with insurance) output units where applicable. Therefore, we remove 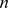 from the theoretical model when moving to an applied model. In addition, due to the differences in age structure among populations, we age-standardize the hospitalization rates (*U*, 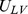, and 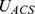). As a result, *Ag* is removed from the theoretical model. In addition, the access-related variables, *Ap* and *Am*, are removed from the model for the following reasons: 1) Acceptability was defined by Penchansky and Thomas [23] as capturing the religious or racial/ethnic fit between a person and the health care facilities, thus is very likely outdated and 2) Accommodation attempts to account for waiting times, hours of operation, telephone appointment systems, and other non-supply related factors of the health care facility. These factors should be quite constant among modern hospitals. We specify a full applied model of hospital utilization as:
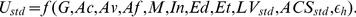
(6)


We evaluate the applied model using linear regression techniques, thereby allowing the potential effect of Roemer’s Law to be identified through the observed relationship between hospital bed availability (*Av*) and hospital utilization (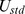). In the regression model, the coefficient value for *Av* defines the independent nature of this relationship, considering that differences in utilization among populations due to size, demographic structure, insurance coverage, and health status are accounted and potential confounding from LV and ACS hospitalizations are also considered.

The proposed framework is implemented in an explicitly spatial context, acknowledging the role of geography in interactions among populations and hospitals. First, because all populations do not have equivalent geographic access to the same hospital services, we incorporate the spatial character of hospital utilization by limiting our analysis to only those hospitalizations where services were demanded locally. Second, we overcome incomplete measures of hospital bed availability by calculating a metric that captures the interaction between distance, hospital bed supply, and demand. Third, we employ spatial regression models which incorporate the spatial structure of the proposed framework, thus counteracting the problems associated with spatial autocorrelation.

The nature of the available data requires that we examine Roemer’s Law at an ecological level. Because the data are aggregated to population units, we address issues stemming from the Modifiable Areal Unit Problem (MAUP) [35], [36]. The MAUP arises when correlation or regression-based analysis are influenced by the particular spatial resolution or zoning scheme of the data. In extreme cases, regression coefficients may flip from positive to negative or statistical significance may be greatly altered when models are built at an alternate scale or with an alternate zoning scheme [37]–[39]. Therefore, we explore the stability of Roemer’s law by evaluating the relationship between hospital bed availability and hospital utilization over multiple levels of data aggregation.

## Materials and Methods

### Study Area

Our case study explores the relationship between hospital bed availability and utilization for the state of Michigan. As of 2010, Michigan had a population of 9,883,640 residents served by 169 acute care hospitals with 26,180 total licensed inpatient beds. In 2010, there were 1,127,576 hospital admissions of Michigan residents to Michigan hospitals and a total of 5,313,149 days spent in hospitals, resulting in an overall patient day utilization rate of 0.537 patient days per person. For every 1,000 people, there were 9.51 hospital admissions per month, which is slightly higher than the national averages of 8 per 1,000 found by Green et al. [40] and 9 per 1,000 as reported by White et al. [41].

**Figure 1:**
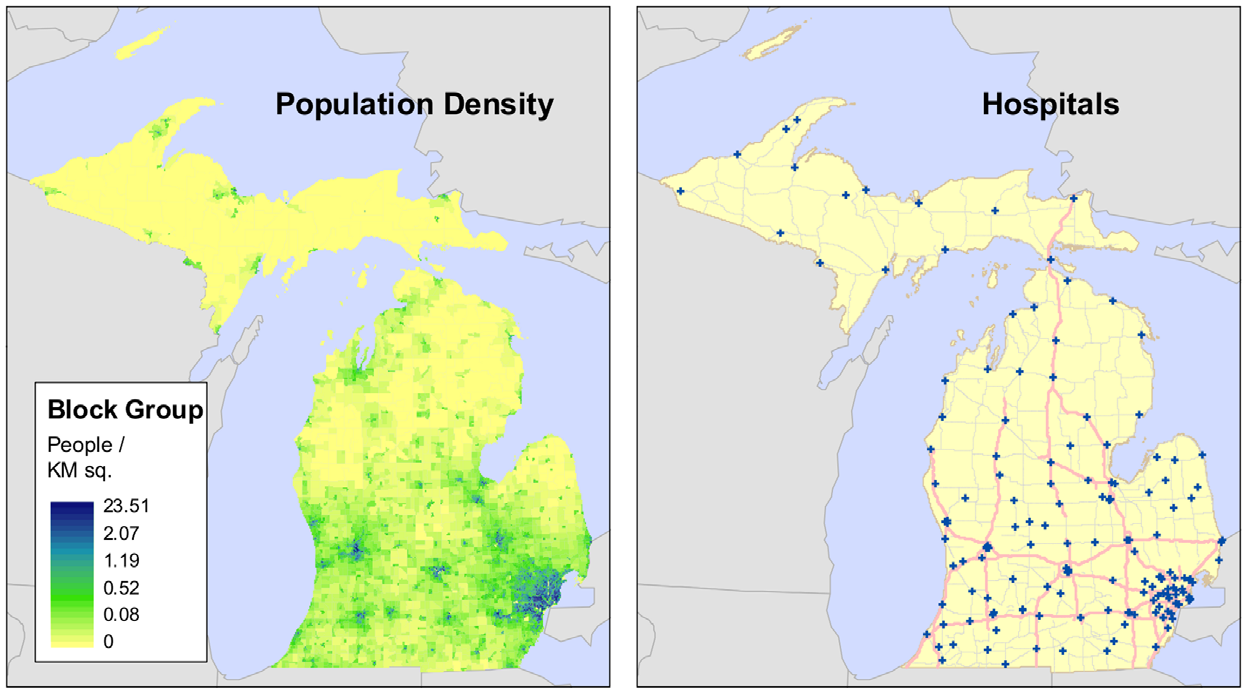
Population distribution and hospital locations in Michigan.

Michigan employs a CON program to regulate the availability of inpatient hospital beds [42]. To assess the needs of the population, a bed need methodology is implemented to predict the future demand for hospital beds, which is compared with current levels of supply [43]. Michigan serves as a satisfactory test case due to the large number of hospitalizations and population, the state’s relatively stable system of acute care hospitals, and a diverse collection of rural and urban areas with varying population densities, health care services distributions, and demographic characteristics (see Figure 1) by which to examine Roemer’s Law.

### Population Data

The Zip Code boundary data used for Michigan were acquired from the ESRI ArcGIS v10 data CD. Prior to the analysis, the 908 unique Zip Codes were aggregated into 895 Zip Codes due to mismatches between the spatial data and the hospital utilization data. The 2010 population and demographic attribute data were acquired from the US Census Bureau (http://2010.census.gov). Block-level data for age (*Ag*), gender (*G*), race/ethnicity (*Et*) were aggregated to their respective Zip Code boundaries. The age-specific data were aggregated into 5 year categories for 0 to 84 years of age with an additional category for 85 and older. Income (*In*), education (*Ed*), and mobility (*M*) attributes were culled from the 2006–2010 American Community Survey 5-year estimates (http://www.census.gov/acs/www/). These data are available at the block group level and were aggregated to the Zip Code boundaries. A small number of block group values were not reported (48 blocks with a population of 52,593, roughly 0.5% of the total state population). Values for the missing block group data were estimated using a weighted average of first-order (queen’s case) neighboring values [44]. First-order neighbors are defined as areas sharing a common boundary. 2009 Small Area Health Insurance Estimates (SAHIE, http://www.census.gov/did/www/sahie/) data were used for health insurance rates (*Af*). For this analysis, we only considered the health insurance status of people under 65 years of age. Because SAHIE data are only available at the county level, Zip Code-level data were estimated using the age-specific rates found in the SAHIE data and age-specific population distribution of the Zip Codes.

### Travel Time Data

Travel time data were derived using a custom-built GIS road network model. The most recently available roads database (2009 version 10a, http://www.michigan.gov/cgi) was downloaded from the Michigan Center for Geographic Information and used to construct the network travel model. Travel speeds for each road were assigned using the road attribute data and a hierarchical speed limit classification system [45].

### Ethics Statement

The Michigan Hospital Inpatient Database (MIDB) consists of routinely collected information on patient’s hospital discharge for billing purposes. The patients provided written consent for their information to be stored in the hospital database. Because this information is protected by HIPPA rules, all identifiable patient information was removed from the MIDB prior for use in this research. The participants therefore, did not provide their written or verbal informed consent to participate in this study. Written consent was not obtained because identifiable information on patients was not available in the MIBD data used in this research. The Michigan State University Internal Review Board Ethics Committee approved this consent procedure and determined the use of the de-identifiable MIDB data exempt for use in this research (IRB #07-362– April 23, 2012).

### Hospital Utilization Data

Inpatient hospitalization data were gathered from the 2010 MIDB, a comprehensive record of the state’s inpatient hospitalizations. For each non-psychiatric hospital admission excluding normal newborns, the age, principal discharge diagnosis (ICD-9-CM), length of stay in days (LOS), Zip Code of residence, and admitting hospital were collected. Travel time was attached to each discharge, calculated from the population-weighted centroid of the Zip Code of residence and the location of the admitting hospital [46]. Hospitalizations occurring more than 60 minutes from the patient’s residence were removed from the analysis. This geographic constraint accounts for two scenarios in which hospitalizations would not be affected by the hospital bed availability of nearby hospitals, thus confounding the analysis. First, it removes hospitalizations where patients traveled a long distance due to the availability of hospital-specific *services*, not hospital bed availability. Second, the constraint removes hospitalizations that occurred when the patient was a significant distance away from their residence (e.g., while on vacation) and not affected by local hospital bed availability. While the 60 minute cutoff value is arbitrary, it is based on previous research exploring spatial accessibility in regions having highly rural populations [47]. Of the total patient days in 2010, 93.2% were served by a hospital within 60 minutes of the patient’s residence.

The LV hospitalization (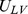) data used in this analysis included discharges for Myocardial Infarction, Ischemic Stroke, and Hip Fracture [48] (ICD-9-CM codes: Myocardial Infarction (410), Ischemic Stroke (431, 434–438), and Hip Fracture (808)). ICD-9-CM codes for the ACS hospitalizations (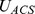) were culled from the Dartmouth Atlas of Healthcare [19]. In 2010, there were 659,997 patient days for ACS conditions and 229,834 for LV conditions.

**Figure 2:**
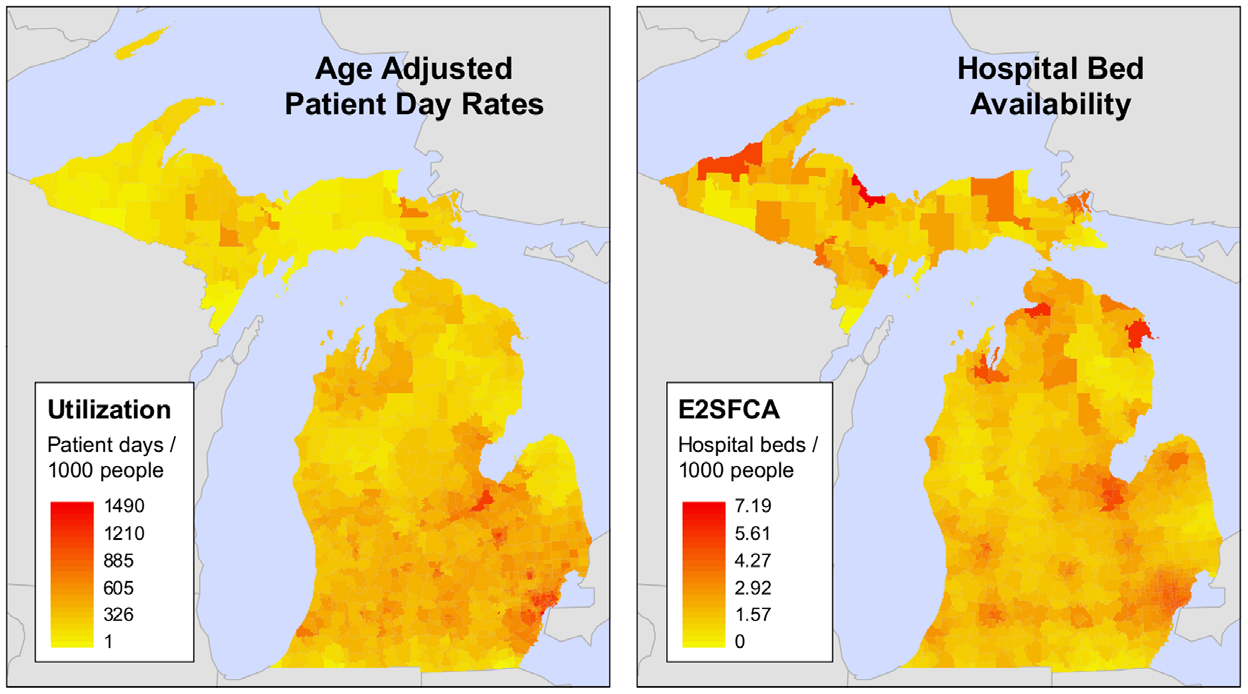
Age adjusted hospital utilization (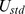) and hospital bed availability (*Av*, E2SFCA) in Michigan.

Because the age distribution of populations is not homogeneous among areal units, the hospitalization data were standardized via the direct method of standardization [49]. Michigan’s 2010 population was used as the standard population. Age standardization was accomplished in a two step process. Some of the state’s Zip Codes contain small populations in each age-specific category and thus violate the 20/50 rule for calculating health-related incidence rates [50]. In addition, as previously mentioned, inpatient hospitalizations are also subject to random fluctuations of ill-health events. Therefore, the first step in the age standardization process was to calculate each areal unit’s age-specific patient day usage rates using an local Empirical Bayes (EB) smoothing method [51]. This smoothing method assumes that the patient day count data follow a Poisson distribution, while also borrowing strength from the patient days and populations of neighboring regions [44], [52]. The neighborhood structure for the EB smoothing process was defined via first-order neighbors. Once the age-specific rates were smoothed, each areal unit’s age-specific patient day rates were multiplied by the age-specific distribution of Michigan’s population. To calculate the final standardized hospital utilization rates (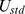, 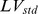, and 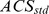), the age-specific data were summed and divided by the total state population (see Figure 2).

Following the age-standardization process, the hospital utilization rate data (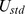) were converted to a Standardized Rate Difference by subtracting the average utilization rate of the entire state from the age-adjusted utilization rate of each observation. This was a simple scalar transformation allowed for improved interpretation of the results such that observations with rates greater than 0 are higher than the state average and those less than 0 are lower.

### Hospital Bed Availability

A comprehensive and robust measure of hospital bed availability (*Av*) is necessary to properly examine Roemer’s Law. However, as previously stated, characterizing the availability of hospital beds to populations can be a complex task. The complexity arises from the intrinsic coupling of availability (supply and demand) and accessibility (*Ac*, distance) that defines the quantity of opportunities that can be considered available. In previous studies of Roemer’s Law, container-based metrics have been commonly employed. These metrics assign the supply of hospital beds to a population unit when the hospital is located within the geographic boundaries of the unit. The number of beds located within the unit is then divided by the unit’s population, producing a beds per person rate. Although container-based metrics are easy to understand and provide highly interpretable output units, they ignore the important accessibility component by omitting the ability to travel outside of the population unit. As a result, *Ac* is not explicitly considered in container-based measures.

The integration of availability and accessibility has been deemed “spatial accessibility” [17]. Spatial accessibility metrics consider the intertwined nature of supply, demand, and distance. We employed a spatial accessibility metric, the enhanced two-step floating catchment area (E2SFCA) [53], to measure the availability of hospital beds. The E2SFCA overcomes the theoretical limitations of container-based measures by allowing catchment areas for supply and demand locations to “float” based on travel distance or travel time in lieu of adherence to administrative boundaries. As a result both *Ac* and *Av* are considered simultaneously.

The E2SFCA is based on a gravity model wherein the theory of distance decay – the probability that utilization will decrease with increased distance – is implemented through a set of “weight values”. Gravity-based models are generally limited by an arbitrary selection of a distance decay function and 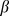 parameter describing the magnitude of decay [54]. However, because the actual travel patterns of Michigan residents are known, our study is not limited by this arbitrary selection process. Using all hospitalizations in Michigan, we calculated the cumulative probability of patient day utilization by distance (measured as travel time) to hospitals. These data were employed to create a model that provided weight values for the E2SFCA. The empirical cumulative probability (*C*) and travel time (*d*) data were used to estimate the parameters of the downward log-logistic decay function [55]:
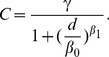
(7)


**Figure 3:**
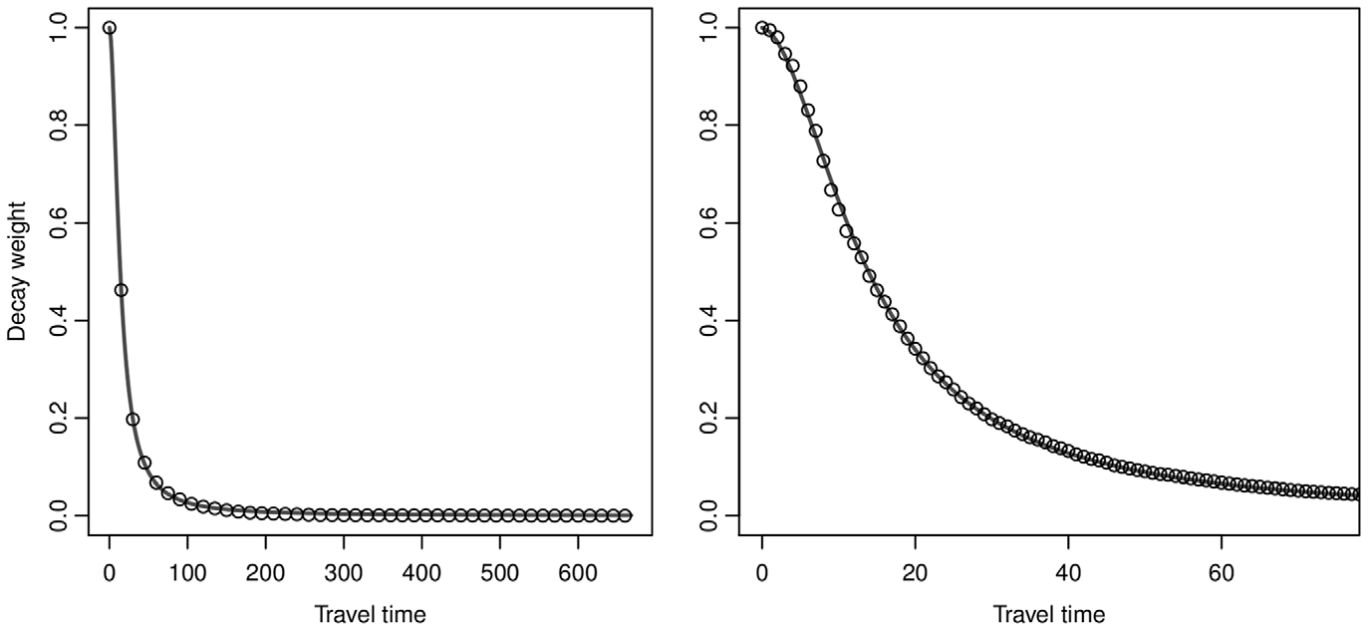
Distance decay of hospital utilization in Michigan. Left: The entire range of the inpatient travel data. Right: A subset of the travel data. The circles are the cumulative proportion of patient day utilization (data are thinned for display purposes) and the line is the downward log-logistic function fit to the data.

In this function, 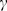, 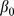, and 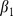 are the parameters to be estimated. The 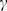 parameter controls *C* at 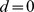; because *C* must equal 1 at 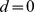 (all hospitalizations occurred at a hospital more than 0 miles from the patient’s residence), we were able to simplify the parameter estimation process by fixing 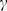 to 1. The 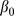 and 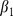 parameters were estimated using the non-linear least squares estimator available in **R** [56]. The resulting parameter values were 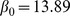 and 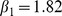. Both parameters were statistically significant (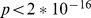) and the model produced a low residual standard error (RSE = 0.003) with an excellent curve fit (see Figure 3).

In the first step in the E2SFCA, the supply ratio is calculated at each facility. Using the network dataset, travel time rings were created for each hospital at 5 minute intervals to a maximum of 45 minutes and a final ring was created from 45 to 60 minutes to incorporate travel in the rural regions in the state [47]. A weight value (*W*) was assigned to each travel ring using the downward log-likelihood function and the travel time values comprising the ring (see Table 1, the sets 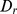 and 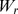). The population data were spatially joined to the travel time rings. The supply (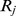, beds/person) is calculated at each facility as follows:
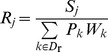
(8)where 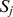 is the number of licensed hospital beds at hospital *j*, 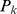 is the population of the unit falling within a travel time ring (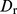), and 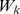 is the specific weight value for the travel time ring (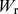) the population unit falls within. Census block centroid points were used in this step as they offered the most accurate representation of population location.

**Table 1:** Weight values for E2SFCA.

Minutes	Weight (*W*)
0–5	0.9459
5–10	0.7544
10–15	0.5511
15–20	0.3993
20–25	0.2957
25–30	0.2253
30–35	0.1765
35–40	0.1417
40–45	0.1161
45–60	0.0832

The weights were estimated from hospital utilization data, using the downward log logistic function.

The second step of the E2SFCA calculates the availability of hospital beds (*Av*) as moderated by distance (*Ac*). Rather than using travel time rings, we completed this step using the measured travel time from the population weighted Zip Code centroids to the hospitals (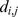), thus calculating the availability of hospital beds at the Zip Code level. This step is formalized as:
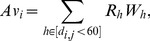
(9)where 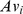 is the availability of hospital beds at population unit *i*, *h* is the set of hospitals within 60 minutes of population unit *i*, 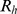 is the supply ratio at each hospital, and 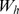 is the weight value calculated using using Eq. 7 and the travel time from unit *i* to hospital *j* (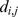).

The E2SFCA provides a measure of hospital bed availability in a beds per person ratio for each population unit (see Figure 2). Although the E2SFCA output is defined as an “availability” metric and labeled *Av*, we stress that the metric integrates both *Av* and *Ac* in its formulation, and thus characterizes spatial accessibility. A more detailed description of the E2SFCA, along with a short worked example, is provided in Text S1.

### Clustering Methodology

Much of the available literature regarding data aggregation in health services research pertains to the creation of *small-areas* for investigating health disparities among regions, e.g. [57]. Generally speaking, these methods use demographic characteristics of the initial areas to create clusters of homogeneous, contiguous regions [58]. Although a number of methods have been proposed for creating small-areas, these were deemed inappropriate for our study. Specifically, we believe that implementing a method that clusters the areal units by the same attributes that were being used to explore Roemer’s Law would essentially be optimizing the aggregation process to achieve a stronger statistical outcome [35]. Hence, the level of objectivity in our test of the MAUP would be diminished [59].

Given this problem, we implemented a clustering methodology that incorporates hospital utilization patterns and geographic location, identifying geographically promixal areal units whose populations use a similar set of hospitals [60]. The resulting clusters are based on similarities in hospital use; however, they are not explicitly optimized based on the same population attributes used to construct the regression models. Essentially, the clustering methodology is based on principles garnered from small-area studies, but does not produce the statistical bias likely present when using the same set of attributes for the purpose of grouping the data *and* constructing the regression models.

The initial observation units (Zip Codes) were grouped into clusters using the K-means clustering algorithm with rational starting locations provided by Ward’s Hierarchical clustering [61]. We clustered the original Zip Code data based on their hospital utilization patterns and geographic location simultaneously. The utilization pattern data were an *n* × *m* matrix containing the proportion of each Zip Code’s total inpatient hospital days (1:*n*) spent at each hospital (1:*m*), otherwise known as the Commitment Index (CI) [62]. The location of each observation is defined by the travel time from each Zip Code (population weighted centroid) to each hospital, thus comprising another *n* × *m* matrix. Representing geographic location as a set of travel distances, rather than coordinates from a traditional planar coordinate system (e.g., latitude and longitude), allows for factors influencing the true separation among places (i.e., road infrastructure, travel speeds, or the physical landscape) to be more accurately characterized [63]. The travel time data were rescaled to match that of the CI data (0–1) by dividing by the maximum travel time between any Zip Code and hospital pair. The two *n* × *m* matrices were appended to create the final data matrix input to the clustering methodology. By clustering the observation units on both locational data and utilization patters, the resulting clusters were spatially contiguous sets of Zip Codes that use a similar set of hospitals.

**Figure 4:**
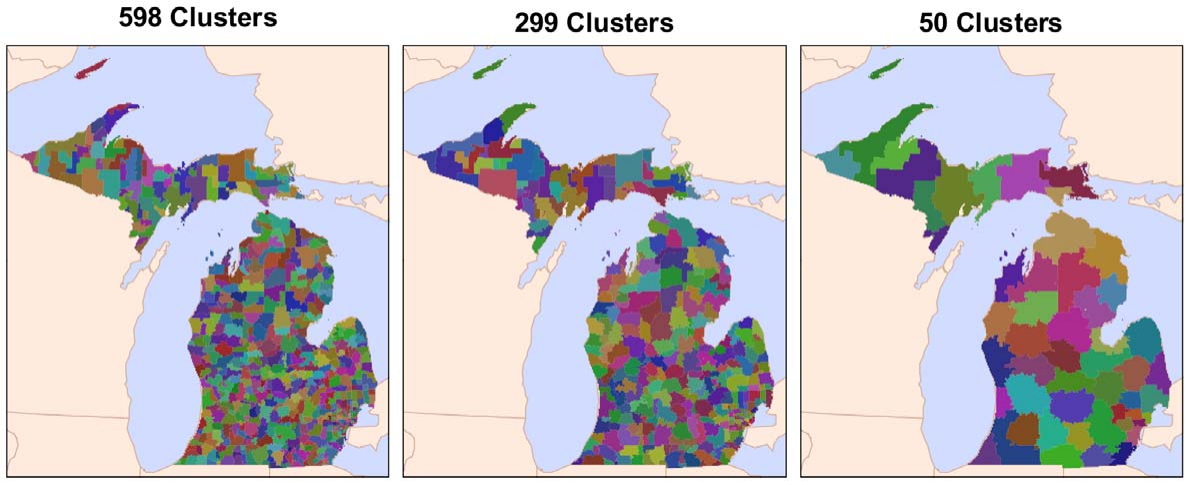
Zip Code cluster examples.

The clustering methodology was run iteratively such that it provided a cluster solution for the set of all possible clusters from 2 to 894 (the set, *S*). We subset the resulting set *S* by implementing a selection method based on the incremental *F* score (*incF*) of each cluster solution [60], [64]. *IncF* measures only the amount of “fit” gained from allowing an additional cluster within the solution, while also penalizing for adding this additional cluster. Local maxima in the *incF* scores represent cluster solutions that provide an substantial improvement in the fit when compared with its immediate neighbors. From the initial set *S*, 276 cluster solutions had local maxima in *incF* scores. These solutions were selected as the aggregation schemes for the regression analysis (see Figure S1 and Table S1). Figure 4 provides three example maps from the final set of cluster solutions. In each aggregation scheme, the attribute data of the Zip Code observation units were aggregated based on their cluster membership. We added the non-clustered units (with the 895 Zip Code observations) as an aggregation scheme for a final set of 277 levels of aggregation.

### Methods to Remove Multicollinearity

Roemer’s Law is examined via inference on the coefficient values from a multiple regression analysis. In multiple regression, multicollinearity arises due to the presence of correlation within the independent variable set, invalidating the modeled coefficient values. Preliminary tests revealed substantial correlation (Pearson’s Correlation Coefficient, 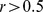) among our independent variables. Given these findings, multicollinearity was addressed using a suite of methods described in the following sections.

#### Principal components analysis

We performed a Principal Components Analysis (PCA) on functional “sets” of variables: income/education, ethnicity/race, transportation, and mobility. Additionally, LV and ACS hospitalization rates were highly correlated with one another; therefore, we identified a fifth functional set, entitled case mix.

By producing uncorrelated component variables, PCA reduces the number of independent variables without a large reduction in the explanatory power of the independent variable set [65]. For example, at most scales of data aggregation, the seven variables within the income/education variable set yielded only a single component. Rather than attempting to identify which of the seven variables would be included in the regression analysis, we were able to extract a single income/education component that sufficiently described the entire suite of variables [66], [67]. Because the data were not standardized, we used the correlation matrix for the PCA [65]. We employed a varimax rotation of the results to assist in interpretation of the component structure [68].

General methods to determine the number of components to extract include manual interpretation of the results or “rules of thumb” [69], thus were not applicable for our study given the large number of PCA runs that were necessary to complete the multi-scale analysis. We implemented a heuristic that allowed for automation of the process to select the number of components extracted [unpublished data]. We added a randomly generated variable to each of the variable sets included in the PCA analysis and generated components. Because PCA provides the loadings on each component for each input variable, the component most heavily influenced by randomness was identified. The PCA was then reimplemented without the random variable, extracting only those components describing more variation in the data than randomness.

The functional sets of variables, the initial input variables, and the interpreted output of the PCA are as follows (see Table S2 for detailed information including the number of components extracted and the amount of variation captured by the extracted components for each functional variable set at each level of data aggregation):

- **Income/education**
*Input variables*
    1 Median household income
    2 Median earnings (16+)
    3 % less than high school education (25+)
    4 % with high school eduction (25+)
    5 % with associates degree (25+)
    6 % with bachelors degree (25+)
    7 % with graduate degree (25+)
*Components*
    1 Income and education (*SES*): High scores reflect populations with higher education, income, and earnings – In 19 of the 277 levels of aggregation, 2 components were identified: one with high scores on education and another with high scores on income and earnings (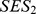). The impacts of this split are noted in the Results section.
- **Ethnicity/race**
*Input variables*
    1 % White
    2 % African American
    3 % Hispanic
    4 % Asian
    5 % American Indian or Alaskan Native (AIAN)
    6 % Hawaiian or Pacific Islander (HWPI)
*Components*
    1 Black/White (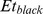): High scores reflect populations with higher proportions of African Americans and lower proportions of Whites
    2–5. Minority population components: High scores reflect observations with higher proportions of Hispanic (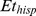), Asian (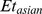), AIAN (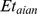), and HWPI (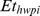) populations – The number of components identified for ethnicity/race were highly variable across the levels of aggregation. The breakdown was as follows: 1 component (32), 2 components (127), 3 components (64), 4 components (52), 5 components (2). The component interpretations are noted in the Results section.
- **Means of Transportation to Work (Mobility)**
*Input variables*
    1 % Automobile (16+)
    2 % Car pool (16+)
    3 % Public transportation (16+)
    4 % Motorcycle (16+)
    5 % Walk, Bicycle, other (16+)
*Components*
    1 Transportation (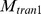): High scores reflect populations that are less reliant on automobiles as the means for their journey to work
    2 Shared transportation (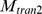): High scores reflect populations with a larger number of people using car pools for their journey to work – In 37 of the 277 levels of aggregation, only a single component was identified: high scores on non-automobile means of transportation. The component 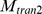 was not included in the final regression analysis as we did not believe that a sufficient theoretical relationship existed between populations with a higher proportion of carpoolers and hospital utilization.
- **Average Travel Time to Work (Mobility)**
*Input variables*
    1 % 0–9 minutes (16+)
    2 % 10–19 minutes (16+)
    3 % 20–29 minutes (16+)
    4 % 30–39 minutes (16+)
    5 % 40–59 minutes (16+)
    6 % 60–89 minutes (16+)
*Components*
    1 High mobility (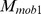): High scores reflect populations that have a higher proportion of long distance (greater than 40 minute) commuters
    2 Medium mobility (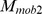): High scores reflect population that have a higher proportion of medium distance (20–40 minute) commuters and a lower proportion of short distance (less than 10 minutes) commuters
- **Hospitalization Case Mix**
*Input variables*
    1 Age-adjusted rate of LV hospitalizations
    2 Age-adjusted rate of ACS hospitalizations
*Components*
    1 Case mix (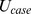): High scores reflect populations that have higher rates of both LV and ACS hospitalizations

#### Bivariate regressions

Because we were interested in the individual impacts of *Av* and *Af* on hospital utilization, these variables (E2SFCA and % population with health insurance) were held out of the PCA analysis. However, we found that *Av* was moderately correlated with the African American population component (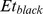) and *Af* was moderately correlated with the *SES* component. In addition, the case mix component (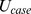) was also correlated with the African American population component (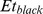). Although the moderate correlation would not invalidate the regression results, we wanted to identify the isolated effects of these variables. Therefore, we adopted the strategy of regressing the variable of interest on its associated correlated variable and using the residuals for further analysis [66]. In this, the residuals function as the “unexplained” portion of the variable of interest, allowing *both* variables to be included in the final model. For example, the variable *Av* becomes the availability of hospital beds *not* associated with 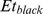. This process was completed for the three noted variables independently at all levels of aggregation when *r* was greater than 0.4. The *F* scores of the overall model and coefficients were tested to ensure the linear models provided significant (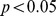) results.

#### Test variance inflation factor

After the principal components analysis and bivariate regressions, we calculated the variance inflation factor (VIF) for the resulting set of independent variables (see Table 2), removing those with a 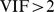 [66]. The variables were removed in a reverse step-wise fashion starting with those considered the least established predictors of hospital utilization toward the most. As the level of aggregation increased and the number of observations became smaller, correlation among the independent variables increased substantially. As a result, we did not perform any subsequent analysis at scales of aggregation with fewer than 37 clusters/observations.

**Table 2:** Attribute variable set.

Input data		TM1	TM2		TM3	Description
Hospital utilization		*U*	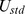		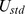	Age standardized hospital utilization rate (normalized to the state’s age standardized rate)
Age distribution of population		*P*	*Ag*			Used to standardize hospital utilization rates
Female population %		*P*	*G*		*G*	
E2SFCA		*A*	*Ac*, *Av*		*Av*	Hospital bed availability not explained by 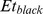
% of population with health insurance (under 65 years of age)		*A*	*Af*		*Af*	Insurance status not explained by *SES*
% Automobile (16+)		*A*	*M*		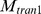	Non-automobile reliant component
% Car pool (16+)						
% Public trans. (16+)						
% Motorcycle (16+)						
% Walk, Bicycle, other (16+)						
% 0–9 minutes (16+)		*A*	*M*		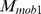 , 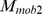	Long commutes to work component, Medium commutes to work component
% 10–19 minutes (16+)						
% 20–29 minutes (16+)						
% 30–39 minutes (16+)						
% 40–59 minutes (16+)						
% 60–89 minutes (16+)						
Median household income		*N*	*In*		*SES*	High income and education component
Median earnings (16+)						
% with less than HS education (25+)		*N*	*Ed*			
% with HS eduction (25+)						
% with associates degree (25+)						
% with bachelors degree (25+)						
% with graduate degree (25+)						
% African American		*N*	*Et*		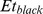	African Am. component
% Hispanic					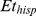	Hispanic component
% Asian					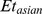	Asian component
% AIAN					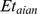	AIAN component
% HWPI					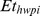	HWPI component
ACS hospitalizations		*N*	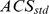		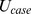	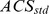 and 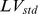
LV hospitalizations			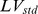			component not explained by 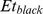

TM1 contains the variable labels in the modified Andersen model from Eqs. 2–4, TM2 contains the variable label from the full model specified in Eq. 6, TM3 contains the final labels used after data processing steps were completed.

### Regression Models

As noted earlier, previous studies of the effects of Roemer’s Law have not incorporated spatial structure. The main implication of this particular model misspecification is that regression coefficients may have contained artificially low standard errors, leading to the rejection of the null hypothesis when it should have been accepted. Initial tests of non-spatial linear models showed high spatial autocorrelation in the residuals with first-order neighboring values (see Figure S2). To account for this phenomena, we used two sets of spatial error models [70], Simultaneous and Conditional Autoregressive Regression models (SAR and CAR, respectively). Both models use the general form,
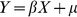
(10)where



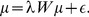
(11)In the spatial error model, *Y* is a vector of 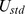 observations; *X* and 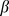 are matrices of independent variables and coefficients, respectively; 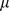 is a vector of autocorrelated residuals; 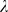 is the autoregressive coefficient; *W* is a neighborhood weight matrix; and 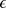 is a vector of non-autocorrelated residuals. The final model can be represented as:
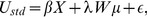
(12)where:



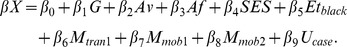
(13)For display purposes, we only include 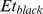 as the other ethnicity/race components were present in far fewer levels of aggregation.

SAR and CAR models differ in their treatment of the spatial pattern in the dependent variable [37], [71]. In the SAR model, the spatial pattern is explained only by the independent variables, simultaneously over all observations. The CAR model uses the independent variables to explain the spatial pattern of the dependent variable, but also conditions the value of the dependent variable on its neighboring values [71]. For all regression models, we defined 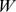 as first-order neighbors. No prior information in our data suggested whether the SAR or the CAR model were more appropriate for this analysis. Additionally, we were unable to locate past research that provided compelling justification for the use of one over the other.

Although we used rate-based data, a Levene test confirmed heteroscedasticity in the initial regression models’ residuals due to differing population sizes among areal units [69] (see Figure S4). Therefore, we implemented weighted SAR and CAR models [44], [72] using the inverse of the square root of the population size as the weights. This specification led to a substantial alleviation of the heteroscedasticity in the model residuals (see Figure S5).

We constructed the weighted SAR and CAR regression models at each level of data aggregation specified in the aggregation schemes. At each level of aggregation, an automated stepwise-like process was employed in the regression models to remove independent variables that were insignificant predictors of hospital utilization rate. The initial regression model was constructed and the independent variables were tested for significance (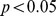). If all variables were significant, the process terminated. If any were insignificant, the variable having the highest *p* value in the model was removed and a new model was constructed. This process continued iteratively until only statistically significant independent variables remained in the final model.

**Figure 5:**
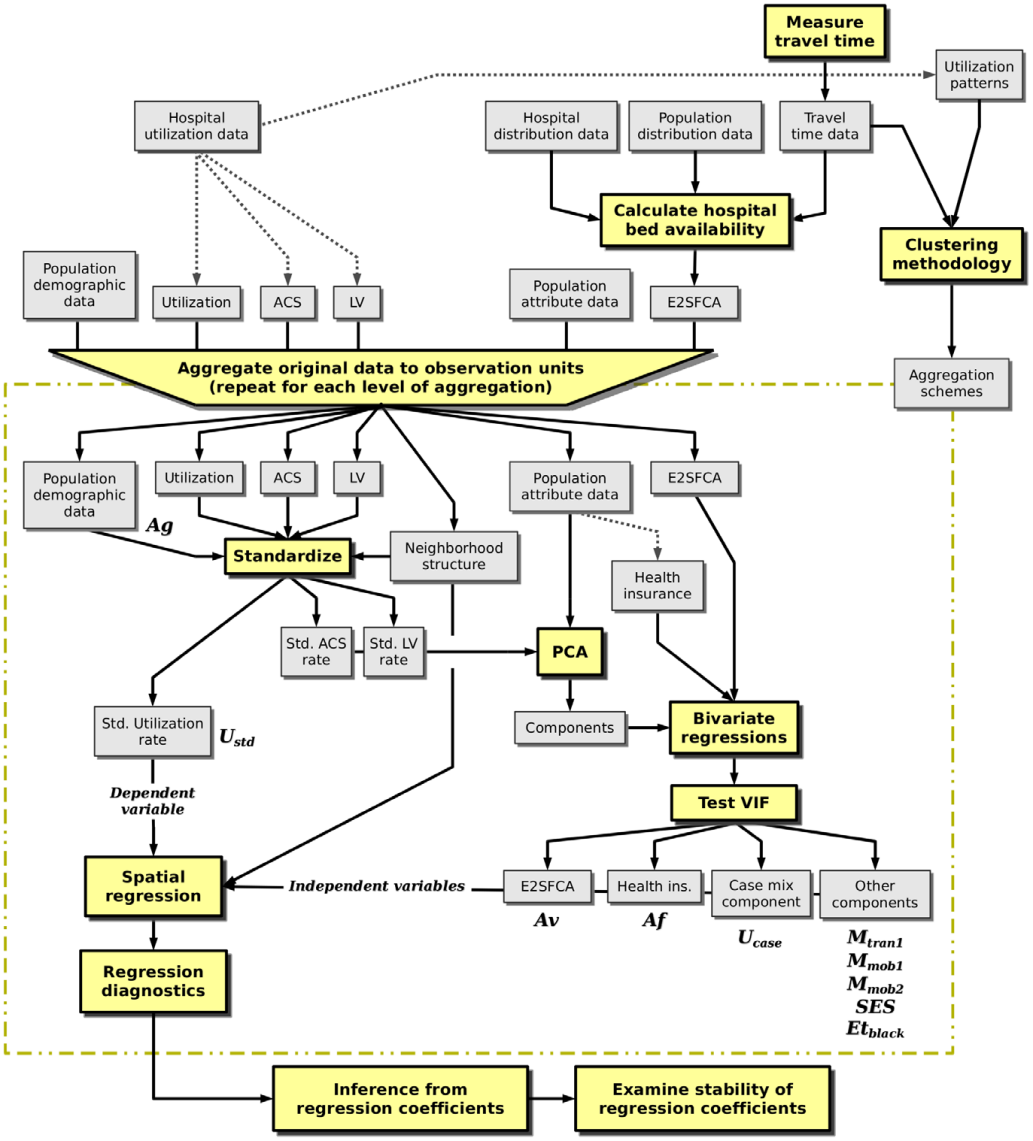
Data and methods workflow diagram. Yellow boxes represent data processing steps, grey boxes represent data, solid arrows represent input/output from data processing, and dotted lines represent a subset process of the respective data.

A comprehensive overview of the research methodology can be found in Figure 5. The figure provides a summary of the techniques employed, along with a workflow of the data processing steps.

## Results

**Figure 6:**
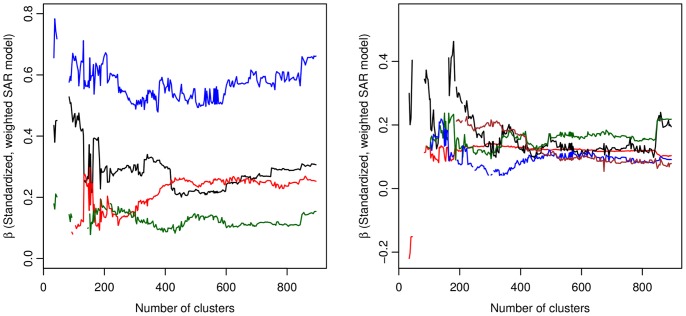
Standardized coefficients for weighted SAR models. Left: *Av* (red), 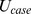 (black), *Af* (green), 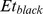 (blue). Right: *SES* (black), 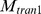 (brown), 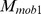 (green), 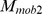 (blue), 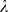 (red). All coefficients are significant at a 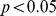.

In total, the SAR and CAR models were constructed at 268 levels of aggregation. In 12 and 31 models for the weighted SAR and CAR models (respectively), the spatial parameter (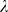) was insignificant and the model considered invalid. The overall coefficient values of the independent variables were very similar among the SAR and CAR models over all levels of aggregation; however, the results of the CAR model contained latent spatial autocorrelation in the residuals at higher levels of aggregation (see Figure S3). Considering these findings, we believe the CAR model was misspecified at these scales of analysis and report only the results of the SAR model. Selected standardized coefficient values for the SAR model are found in Figure 6. Table 3 contains a diagnostic summary of the variables over all levels of aggregation.

**Table 3:** Coefficient statistics.

	weighted SAR model coefficients					
Variable	Total	Model	positive	negative	insignificant	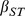
*Av*	268	268	254	0	14	0.213
*Af*	268	268	252	0	16	0.128
*G*	268	254	19	0	235	0.09
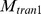	268	248	219	0	29	0.132
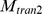	238	0	0	0	0	NA
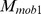	268	260	254	0	6	0.156
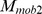	268	252	237	0	15	0.096
*SES*	268	256	256	0	0	0.169
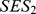	17	17	0	13	4	−0.374
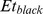	268	268	268	0	0	0.574
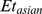	66	63	44	4	15	0.074
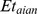	99	98	0	95	3	−0.11
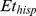	106	103	102	0	1	0.127
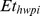	137	137	41	34	62	0.054
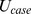	268	268	268	0	0	0.294
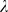	268	268	252	4	12	0.118

Total is the number of times the variable is present; Model is the number of times that the variable was included in the initial model (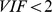); positive is the number of time the variable’s coefficient was significant (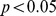) and positive in the final model; negative is the number of time the variable’s coefficient was significant (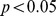) and negative in the final model; insignificant is the number of times the variable’s coefficient was insignificant and removed from the final model; and 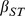 is the mean of the *significant* standardized regression coefficients over all levels of aggregation.

Figure 6 shows that the magnitude of the statistical relationships among the independent variables and hospital utilization rates was quite stable across levels of aggregation. In particular, we find strong evidence of Roemer’s Law as hospital bed availability (*Av*) has a positive, significant relationship with hospital utilization rates (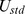) that appears relatively invariant with changes in the scale of the input data. Additionally, LV and ACS hospitalization rates (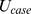), health insurance coverage (*Af*), proportion of African Americans (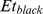), high income and education (*SES*), and higher mobility (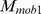 and 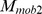) had consistent, positive relationships with hospital utilization rates across levels of aggregation. This *scalar* stability of the coefficients suggests that the observed relationships are process-based, not remnants of a particular scale of analysis nor spurious correlations.

Table 3 provides the mean of the standardized coefficient values (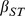) for all the explanatory variables in the SAR models (the mean value was calculated using only statistically significant 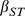 values from the individual models). The results highlight the relative magnitude of each variable’s contribution to variations in hospital utilization rates (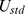). 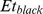 has the greatest affect on 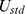 (0.574), nearly two times that of 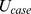, which has the 2nd largest value (0.294). Importantly, these results show that the impact of *Av* on 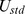 is non-trivial; *Av* has the third largest mean 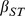 value (0.213), even after removing a portion of the variable’s potential explanatory power through the bivariate regression (due to its correlation with 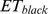). Of the remaining variables that were significant in more than 200 models, higher socio-economic status (*SES*) and higher mobility (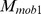) had the largest affect on 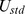 (0.169 and 0.156, respectively). Interestingly, health insurance coverage (*Af*) has a relatively low mean 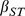 (0.128), although this could likely be a result of diminished explanatory power stemming from the removal of the correlation between *Af* and *SES* (through bivariate regression).

## Discussion

Although Roemer initially seemed somewhat surprised that his statement had been bestowed the status of a *law* [20], our findings provide compelling evidence to support this claim. We found that a positive, significant relationship exists between hospital bed availability and hospital utilization rates while controlling for the most widely accepted determinants of hospital utilization. Additionally, this relationship was consistent across levels of spatial aggregation providing support that the origin of the observed effect is not a product of the scale of analysis.

In previous studies, Alexander et al. [12] and Clark [15] found that hospital beds per capita was not a significant predictor of hospital use rates in Michigan. In Alexander et al., SES variables were the most significant predictors of hospital utilization, whereas board certified physicians and registered nurses per hospital bed were significant predictors in Clark’s study. In contrast, our results illustrate that *both* SES and bed availability have significant impacts on hospital utilization rates; however, we did not consider measures of physicians or nursing as variables in our models. A number of factors cause concern in the results of these previous studies. First, although Alexander et al. controlled for temporal autocorrelation in their regression models, neither study acknowledged the spatial structure of their observations, thus likely misspecifying their regression models. Second, in both studies, hospital bed availability was calculated using a summation of the beds and population within the administrative unit boundaries, not incorporating the travel behavior of patients. Third, both studies were limited to regional-level observation units (58 over Michigan’s lower peninsula for Alexander et al. and 53 over Michigan’s lower peninsula excluding Detroit for Clark) and a single scale of analysis.

As Figure 6 illustrates, in the weighted SAR model, the coefficient for 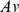 decreases slightly as the data are aggregated to a regional-level scale. The most similar level of aggregation used in our analysis to those employed by Clark and Alexander et al. is 70 clusters (58 observations in the lower peninsula). At this level of aggregation, the weighted SAR model provides a positive, significant coefficient for hospital bed availability; however, the 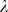 parameter is insignificant in this model. In a non-spatial weighted OLS regression with 70 clusters, we find that hospital bed availability is again not a significant predictor of utilization rates. These results likely stem from the homogenization of the data that occurs as the level of aggregation moves towards this regional scale of analysis. Interestingly, the level of aggregation used by Alexander et al. and Clark is very near an observed threshold where 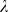 and *Av* become insignificant in the set of SAR models. In fact, at 88 clusters, *Av* is a positive and significant predictor and the 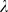 parameter is also significant, suggesting that both Alexander et al. and Clark’s studies may have produced different findings had they used less aggregated data. As a result, the effects of hospital bed availability on utilization rates may go undetected at regional-level scales. More specifically, our results provide empirical evidence of a threshold level in the ability to observe the effects of Roemer’s Law in small area studies.

Recent research has shown the danger in statistical inference garnered from ecological-based relationships at a single geographic scale of analysis. Wright and Ricketts III [39], in a review of Kravet et al. [73], showed that coefficient values related to the supply of health care resources may change in significance and even direction as the scale of analysis changes by way of data aggregation. Their work highlights the problems associated with the MAUP in health-based research. In our study, the stability of the coefficients across levels of aggregation suggest that the observed relationships are not highly susceptible to variation due to the scale in which the data are aggregated. Although levels of aggregation smaller than Zip Codes could not be tested (due to the deidentification of the hospitalization data), the overall statistical strength and invariant nature of the relationship between hospital bed availability and hospital utilization provide strong evidence that our findings are not a product of the MAUP.

With support of Roemer’s Law demonstrated, we turn our attention toward the implications of our research with regards to CON programs. Previous research has suggested that over the past 40 years CON programs have not been successful in controlling health care costs [5], [74], [75]. A recent study by Conover and Sloan [76] reported that Michigan’s CON program had not effectively contained hospital costs and recommended that the state abandon regulation of acute care hospital beds. Although we did not consider the effects of hospital bed availability on health care costs, our findings of do suggest that efforts to control hospital bed availability will affect hospital utilization rates. Furthermore, the significant, stable, and positive nature of the observed relationship indicates that CON-based regulation of hospital bed supply to levels consistent with the needs of the population is justified.

Our results also showed a strong, positive association between a higher proportion of Black (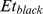) and Hispanic populations (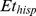) and higher rates of hospital utilization. Given that other possible determinants of hospital utilization, SES and access to primary care (ACS hospitalizations) – which are often associated with contributing to poorer health in disadvantaged populations– were controlled for in our models, these findings are troubling from a social justice perspective. Alarmingly, 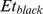 had the greatest effect on hospitalization rates across all levels of aggregation. Although the causes behind these relationships were not further explored in the present analysis, recent work by Grady [77], [78] and Grady et al. [79] has demonstrated that neighborhood segregation is associated with health disparities in New York and Michigan. In the present context, higher hospitalization rates for areas having a higher percentage of Black residents possibly point to underlying health issues that stem from neighborhood effects [80]–[82]. Considering that metropolitan Detroit is one of the most segregated cities in the USA [83] and a large proportion of Michigan’s Black population resides in this region, our findings suggest that a more detailed analysis exploring the effects of race, segregation, and neighborhoods on hospital utilization rates in southeast Michigan is warranted.

Another notable result from this research is the identification of the relative influence that each explanatory variable exerts on hospital utilization rates. Given our efforts to ensure the variables were independent from one another in the regression models and the multi-scale implementation of our research design, the standardized coefficients provide salient information regarding the nature of these relationships. Importantly, 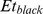, 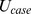, and *Av* are identified as having the greatest effect on hospital utilization rates. Additionally, we find that *SES*, *Av*, and multiple mobility measures impact utilization rates similarly. By revealing the relative magnitudes of these relationships, our results have the potential to guide future public health initiatives aimed toward reducing hospital utilization. Given these ramifications, further examination of the mechanisms leading to the observed relationships is crucial.

### Limitations

Our analysis did not consider alternative neighborhood structures in the EB smoothing process or the spatial regression models. Other neighborhood structures, such as those based on distance or *k*-nearest neighbors, require a defined threshold value for determining neighbor status. Given the large range of data configurations evaluated and their dissimilar geographic scales (for reference, see Figure 4), specifying a single distance or *k* threshold would not provide a consistent spatial structure throughout scales of analysis. Hence, the decision to employ a first-order neighborhood structure was considered necessary due to the multi-scalar nature of the research design. If the neighborhood structure was defined using the 10 nearest neighbors, the neighborhood organization would vary considerably as the data were aggregated to more regional scales (e.g., 10 neighbors may approximate first-order neighbors at low levels of aggregation, but 2nd or 3rd order neighbors at higher levels of aggregation). The same difficultly would manifest if a minimum distance threshold was implemented, augmented by the limitations associated with measuring distances among highly aggregated areal units [84]. For the purposes of our analysis, the first-order neighborhood structure provided a characterization of spatial structure supported by theory [85] and flexible enough to accommodate the multi-scale nature of the research design.

**Figure 7:**
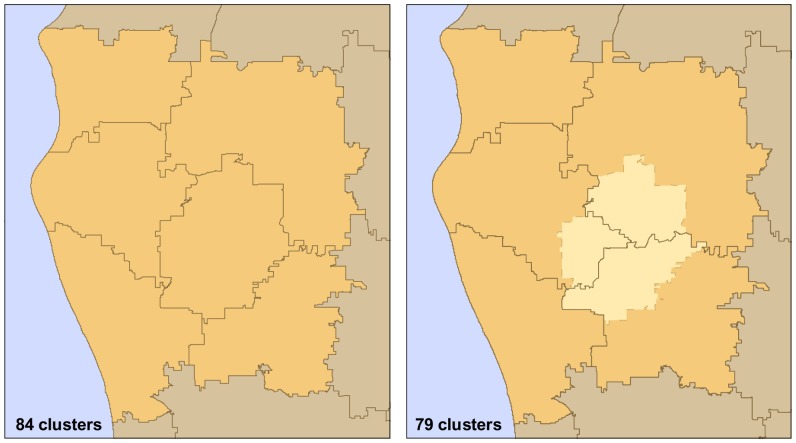
Example of rezoned region. In the 84 cluster solution (Left), the region contains 6 clusters. In the 79 cluster solution (Right), the same region contains 5 non-agglomerative clusters.

Although the scale effect of the MAUP was explored in our analysis, the zoning effect was not explicitly examined. However, the effect of zone modification was implicitly addressed through the use of a non-agglomerative clustering methodology. Specifically, for each iteration in the clustering method, the Zip Code data were clustered, not the clusters from the previous step in the iteration. Hence, in many cases, regions were essentially “rezoned”, thus providing an implied examination of the zoning effect of the MAUP. To illustrate this point, Figure 7 contains an example of a small region that was rezoned rather than agglomerated as the level of aggregation changed. Given this limitation, we recommend that further consideration of the zoning effect of the MAUP to be included in future research of Roemer’s Law.

### Conclusions

The relationship between hospital bed availability and hospital utilization is spatial in nature. Yet, previous studies have not fully considered the spatial issues embedded in Roemer’s Law. This absence led us to question whether “a bed built is a bed filled” is simply a statement or belief that has become entrenched in the lexicon of health services research or an actual process that can be observed.

This research found a positive, significant association between hospital bed availability and hospital utilization rates while controlling for other determinants of hospitalization. The research design was implemented in an explicitly spatial context, incorporating a behavioral model of health care utilization with the spatial and aspatial aspects of health care access and utilization and considering the spatial structure of these relationships. The ecological nature of the research design limits our ability to establish a causal link between hospital bed availability and utilization rates. However, given our research approach, the magnitude and significance of the observed relationship, and the stability of the relationship over levels of data aggregation, we believe we have provided the most compelling evidence to date of the existence of Roemers Law.

Harrington et al. [86] note that a limited number of health services studies have integrated health behavioral models with geographical or spatial factors. In this research, we have not only provided useable and pertinent findings, but also have delivered a research protocol that can be implemented in future health services research.

The outcomes of this study address the research question originally posed; however, they also elicit a number of new issues regarding health care policy and health services research. Perhaps the most important follow up query is, “what are the causal mechanisms that lead to higher hospitalization rates in areas with higher hospital bed availability?”. While some have suggested that the answer lies in the clinical decision-making process of physicians [2], others have suggested that it may be the hospitals themselves [11] and the question remains unanswered.

Recent hospital construction and expansion (bypassing CON regulation through legislative action) and a proposed transfer of beds into areas of the state without a demonstrated need for additional hospital beds highlight the importance of our findings in Michigan. Nationally, as health care systems and hospitals adapt to increasing health care costs, a changing economic climate, and a dynamic regulatory environment due to the Affordable Care Act, understanding of the effects of hospital bed availability on hospital utilization and their causes is more critical than ever.

## Supporting Information

Figure S1 ***incF*** **scores for cluster solutions in set** ***S*** **.** Black points represent peak values in *incF* scores. The data have been truncated for display purposes.
(TIF)

Figure S2 **Moran’s I of regression residuals for weighted OLS regression model.** All values less than 0.05 (dotted line) have significant spatial autocorrelation in the model residuals.
(TIF)

Figure S3 **Moran’s I of regression residuals for weighted SAR and CAR models.** All values less than 0.05 (dotted line) have significant spatial autocorrelation in the model residuals.
(TIF)

Figure S4 **Levene Test of regression residuals for SAR and CAR models.** All values less than 0.05 (dotted line) have significant heteroscedasticity in the model residuals due to population size.
(TIF)

Figure S5 **Levene Test of regression residuals for** ***weighted*** **SAR and CAR models.** All values less than 0.05 (dotted line) have significant heteroscedasticity in the model residuals due to population size.
(TIF)

Table S1 **Cluster solutions and** ***incF*** **scores.**
(PDF)

Table S2 **Number of components and % of the total variance explained for each functional set of variables.**
(PDF)

Text S1 **Enhanced Two-Step Floating Catchment Area.**
(PDF)
