# Supplementary figures and images for: Do More Hospital Beds Lead to Higher Hospitalization Rates? A Spatial Examination of Roemer’s Law
Source: PLoS One. 2013 Feb 13;8(2):e54900. doi: 10.1371/journal.pone.0054900 (PMC3572098; doi:10.1371/journal.pone.0054900)

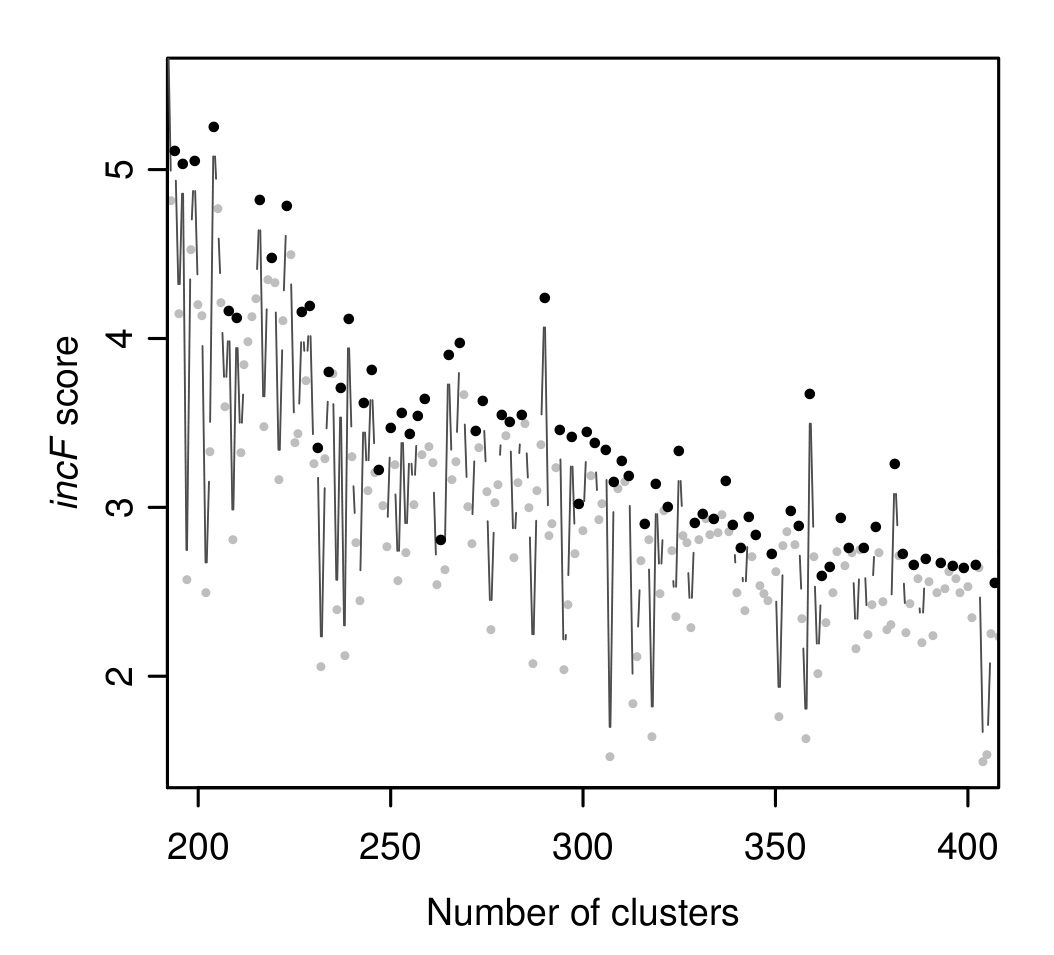

Supplement: Figure S1 — incF scores for cluster solutions in set S . Black points represent peak values in incF scores. The data have been truncated for display purposes. (TIF) [file pone.0054900.s001.tif]

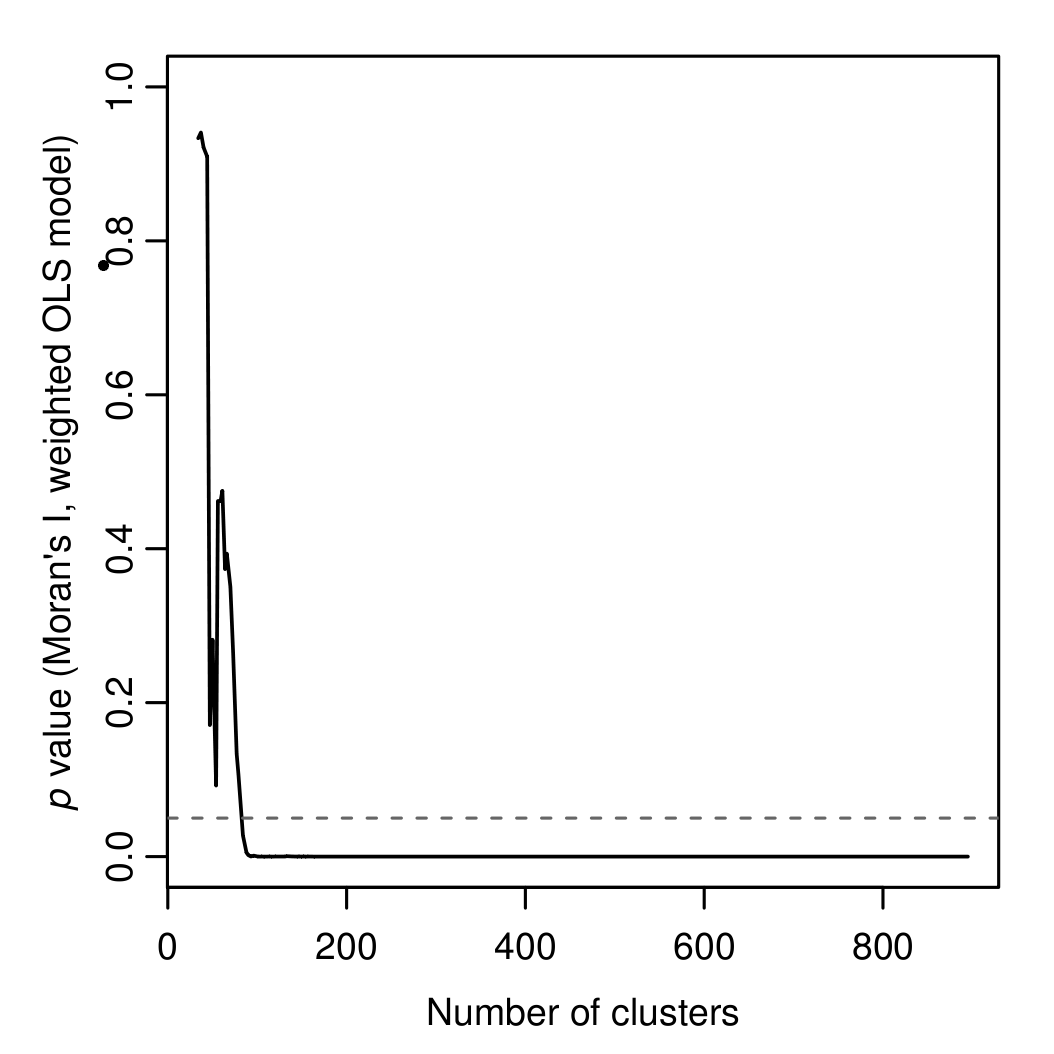

Supplement: Figure S2 — Moran’s I of regression residuals for weighted OLS regression model. All values less than 0.05 (dotted line) have significant spatial autocorrelation in the model residuals. (TIF) [file pone.0054900.s002.tif]

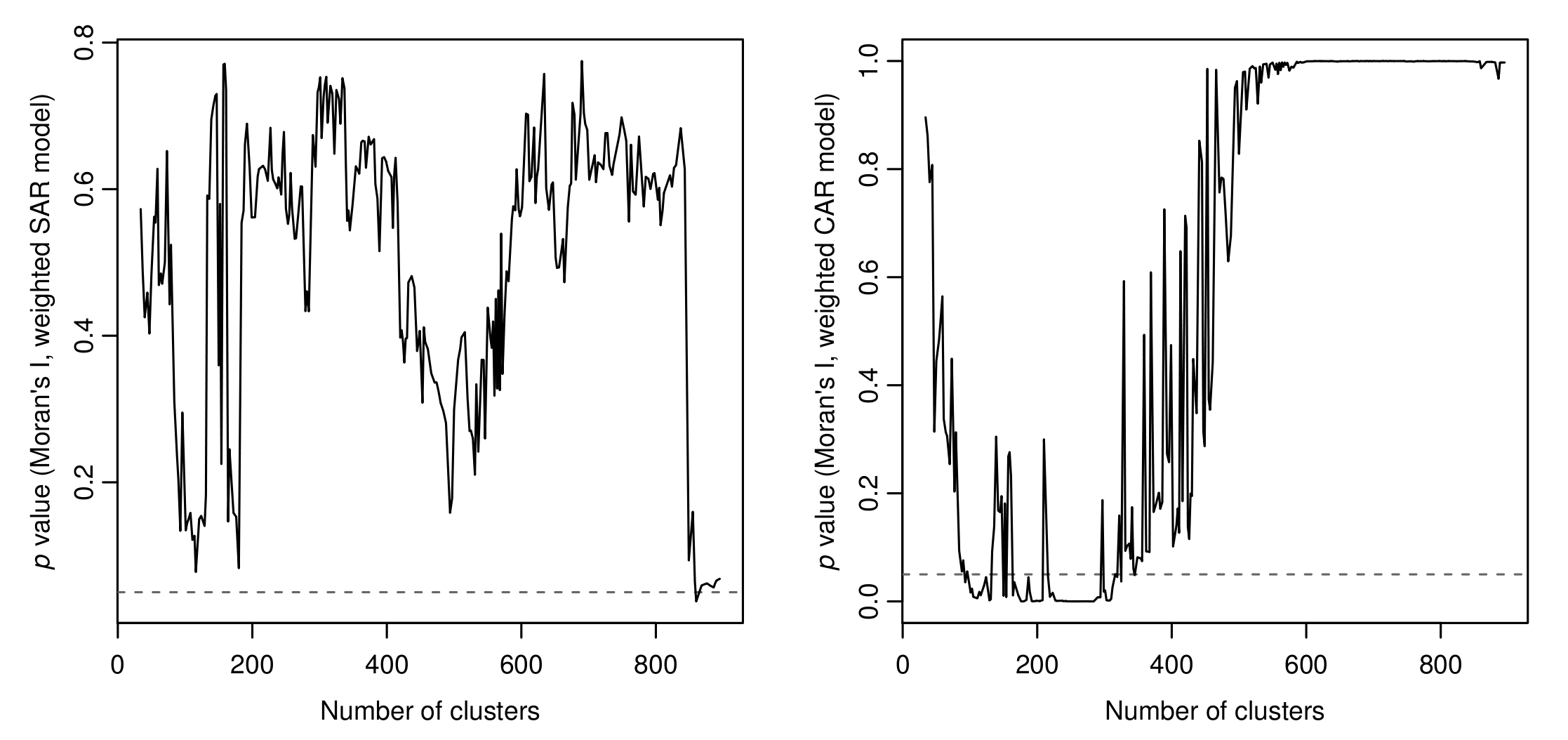

Supplement: Figure S3 — Moran’s I of regression residuals for weighted SAR and CAR models. All values less than 0.05 (dotted line) have significant spatial autocorrelation in the model residuals. (TIF) [file pone.0054900.s003.tif]

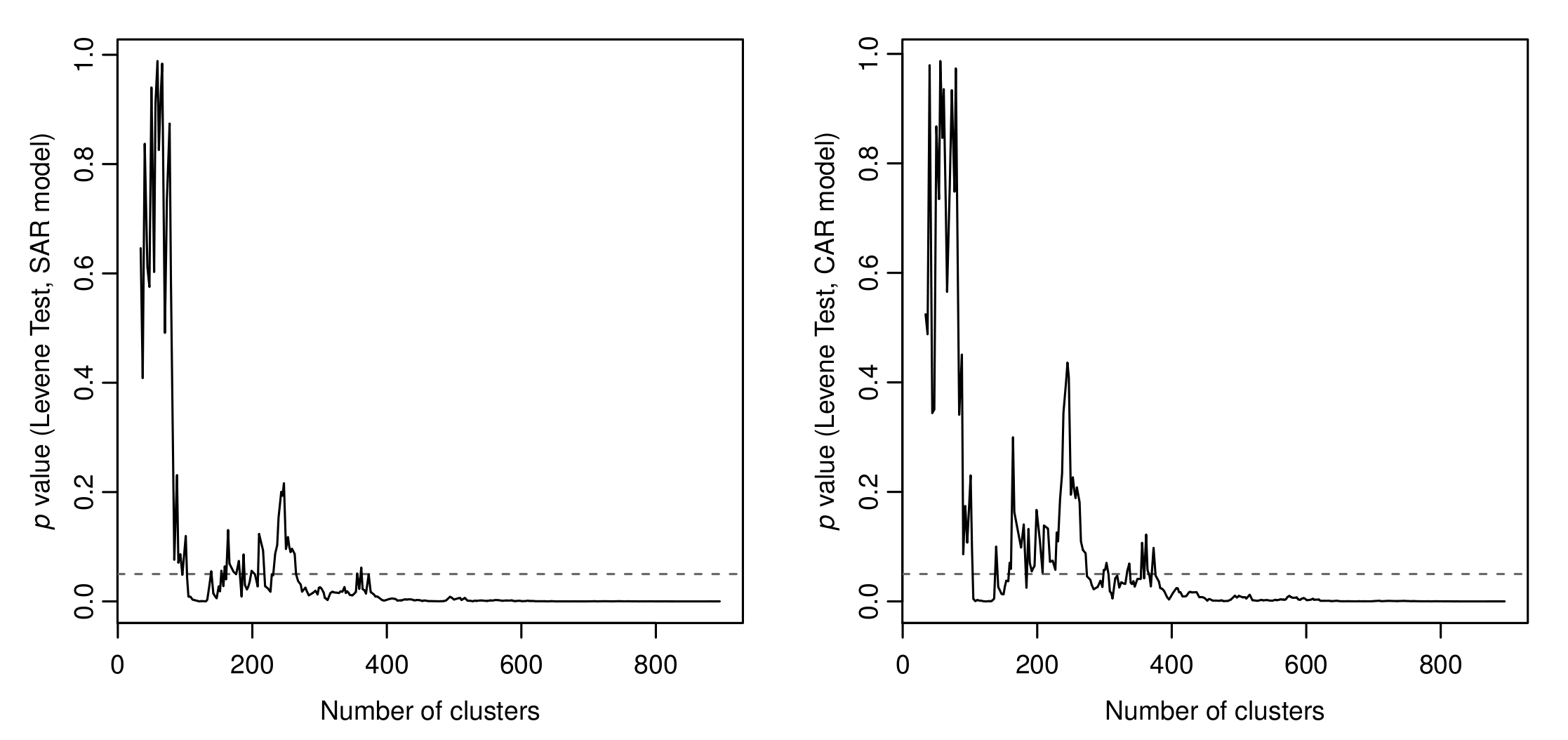

Supplement: Figure S4 — Levene Test of regression residuals for SAR and CAR models. All values less than 0.05 (dotted line) have significant heteroscedasticity in the model residuals due to population size. (TIF) [file pone.0054900.s004.tif]

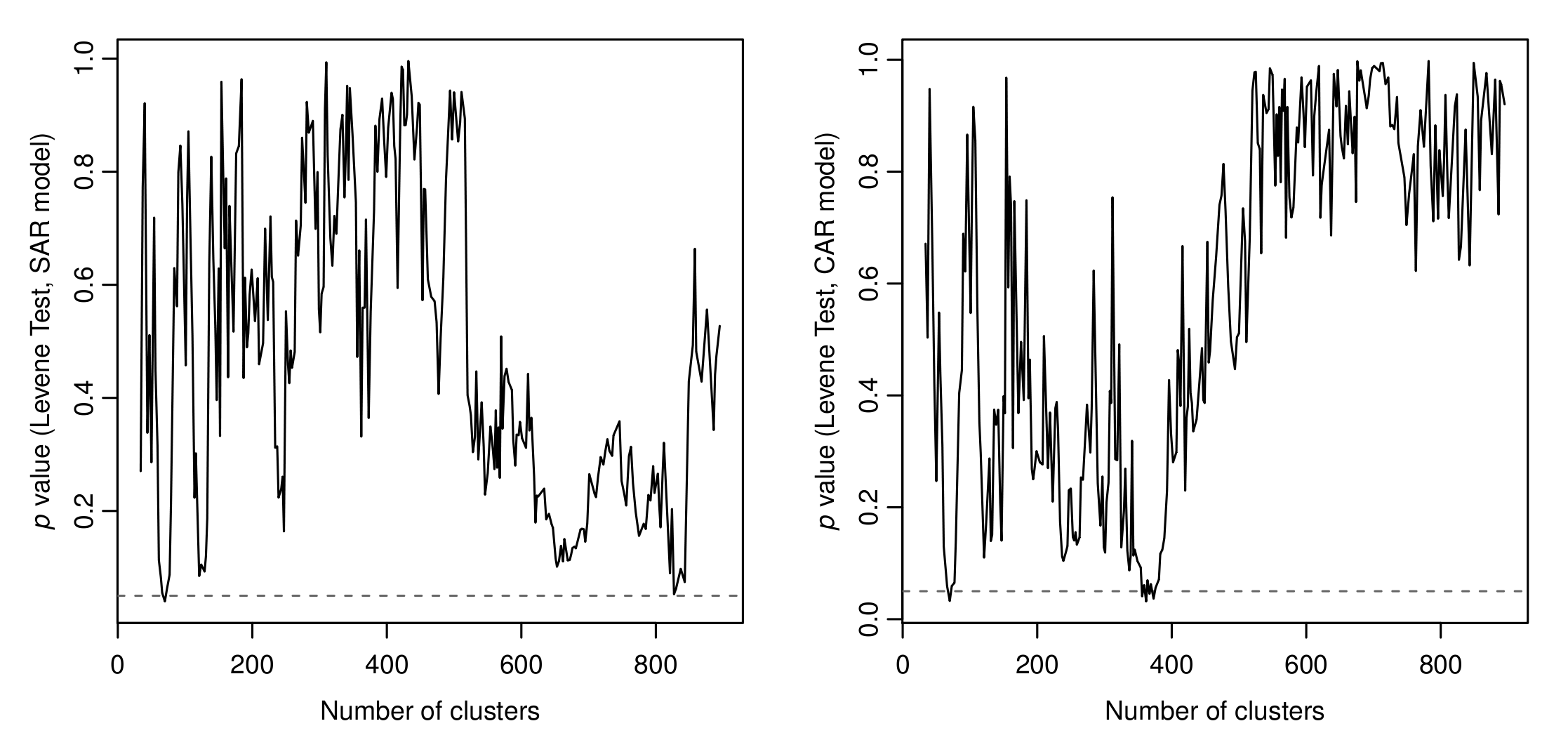

Supplement: Figure S5 — Levene Test of regression residuals for weighted SAR and CAR models. All values less than 0.05 (dotted line) have significant heteroscedasticity in the model residuals due to population size. (TIF) [file pone.0054900.s005.tif]
