# Supplementary material for: Do More Hospital Beds Lead to Higher Hospitalization Rates? A Spatial Examination of Roemer’s Law
Source: PLoS One. 2013 Feb 13;8(2):e54900. doi: 10.1371/journal.pone.0054900 (PMC3572098; doi:10.1371/journal.pone.0054900)
Supplement: Table S1 — Cluster solutions and incF scores. (PDF) [file pone.0054900.s006.pdf]

Table S.1. Cluster solutions and *incF* scores.

CL	*incF*	CL	*incF*	CL	*incF*	CL	*incF*
8	173.588	219	4.478	420	2.574	634	1.950
13	117.188	223	4.785	422	2.525	637	1.936
17	57.137	227	4.155	424	2.514	641	1.968
19	46.520	229	4.191	426	2.470	645	1.957
21	41.105	231	3.352	428	2.446	647	1.928
23	35.267	234	3.802	430	2.422	651	1.915
27	28.022	237	3.709	432	2.421	653	1.896
29	27.952	239	4.119	437	2.399	656	1.890
32	21.648	243	3.621	441	2.409	659	1.876
34	32.543	245	3.812	445	2.388	662	1.869
37	22.762	247	3.220	447	2.343	664	1.901
40	24.243	250	3.472	449	2.370	669	1.887
44	24.131	253	3.558	453	2.367	672	1.882
47	22.427	255	3.436	455	2.367	674	1.865
50	17.827	257	3.541	457	2.362	676	1.838
54	17.783	259	3.644	461	2.584	679	1.839
56	23.635	263	2.805	466	2.379	681	1.850
59	15.538	265	3.905	471	2.305	688	1.787
61	17.436	268	3.972	474	2.347	690	1.787
64	10.838	272	3.450	477	2.350	693	1.779
66	18.027	274	3.630	480	2.330	695	1.771
70	11.505	279	3.544	484	2.287	698	1.769
73	20.162	281	3.508	488	2.295	701	1.773
77	13.521	284	3.550	494	2.287	709	1.799
79	14.299	290	4.243	497	2.284	711	1.784
84	8.937	294	3.461	500	2.287	714	1.789
88	9.763	297	3.419	506	2.408	718	1.806
90	12.296	299	3.022	509	2.042	722	1.803
93	8.184	301	3.446	511	2.206	725	1.765
96	8.975	303	3.384	516	2.270	728	1.770
101	10.237	306	3.340	520	2.112	731	1.789
103	8.573	308	3.149	523	2.193	735	1.734
105	8.618	310	3.277	525	2.203	737	1.699
108	9.590	312	3.186	528	2.317	746	1.752
111	6.621	316	2.903	531	2.176	749	1.683
114	10.148	319	3.137	533	2.164	753	1.681
116	9.488	322	3.003	536	2.156	756	1.697
121	7.840	325	3.333	541	2.139	760	1.679
124	6.302	329	2.908	544	2.148	763	1.680
129	10.391	331	2.958	546	2.636	766	1.682
131	7.376	334	2.929	550	2.133	770	1.680
133	8.357	337	3.159	554	2.146	775	1.670
136	7.806	339	2.895	556	2.150	782	1.672
139	6.168	341	2.760	558	2.153	785	1.735
142	5.989	343	2.942	560	1.958	789	1.658
145	6.427	345	2.834	562	2.149	792	1.654
147	6.223	349	2.725	564	2.182	796	1.597
150	5.386	354	2.978	566	2.177	798	1.593
152	6.834	356	2.891	568	2.151	803	1.562
154	6.671	359	3.673	570	2.101	805	1.564
157	6.621	362	2.597	572	2.152	807	1.533
159	5.148	364	2.648	575	2.150	810	1.528
161	6.301	367	2.936	578	2.143	812	1.589
164	6.079	369	2.762	581	2.191	821	1.544
166	5.838	373	2.758	586	2.096	824	1.521
172	7.055	376	2.884	588	2.098	827	1.532
176	6.552	381	3.257	591	2.097	830	1.480
180	6.088	383	2.725	593	2.170	837	1.515
184	5.825	386	2.659	596	2.063	843	1.484
187	4.415	389	2.697	598	2.110	849	1.463
189	4.086	393	2.673	601	2.078	855	1.415
192	6.036	396	2.651	607	2.080	858	1.361
194	5.112	399	2.638	610	2.054	860	1.373
196	5.035	402	2.658	612	1.916	868	1.418
199	5.050	407	2.551	615	2.038	876	1.546
204	5.255	409	2.736	619	2.029	881	1.600
208	4.161	411	2.895	621	2.037	886	1.751
210	4.120	413	3.031	623	2.098	888	1.764
216	4.818	416	2.443	625	2.057	890	1.665
