# Supplementary material for: Do More Hospital Beds Lead to Higher Hospitalization Rates? A Spatial Examination of Roemer’s Law
Source: PLoS One. 2013 Feb 13;8(2):e54900. doi: 10.1371/journal.pone.0054900 (PMC3572098; doi:10.1371/journal.pone.0054900)
Supplement: Table S2 — Number of components and % of the total variance explained for each functional set of variables. (PDF) [file pone.0054900.s007.pdf]

Table S.2. Number of components and % of the total variance explained for each functional set of variables.

	SES		Et		M_{tran}		$Mmob$		U_{case}			SES		Et		M_{tran}		$Mmob$		U_{case}	
**CL**	**n**	**s**	**n**	**s**	**n**	**s**	**n**	**s**	**n**	**s**	**CL**	**n**	**s**	**n**	**s**	**n**	**s**	**n**	**s**	**n**	**s**
34	1	0.85	1	0.43	1	0.64	2	0.89	1	0.93	247	1	0.72	4	0.87	2	0.88	2	0.81	1	0.88
37	1	0.86	1	0.40	1	0.65	2	0.89	1	0.93	250	1	0.71	2	0.55	2	0.88	2	0.81	1	0.88
40	1	0.85	2	0.61	1	0.64	2	0.89	1	0.93	253	1	0.72	2	0.55	2	0.88	2	0.81	1	0.88
44	1	0.83	5	1.00	2	0.88	2	0.88	1	0.93	255	1	0.72	2	0.55	2	0.88	2	0.80	1	0.88
47	1	0.82	3	0.78	1	0.62	2	0.88	1	0.93	257	1	0.72	2	0.55	2	0.88	2	0.80	1	0.88
50	1	0.81	3	0.77	1	0.64	2	0.88	1	0.93	259	1	0.72	2	0.55	2	0.88	2	0.80	1	0.88
54	1	0.81	3	0.76	2	0.85	2	0.88	1	0.93	263	1	0.72	2	0.55	2	0.88	2	0.80	1	0.88
56	1	0.81	2	0.59	1	0.61	2	0.88	1	0.93	265	1	0.72	2	0.55	2	0.88	2	0.80	1	0.88
59	1	0.80	1	0.38	1	0.60	2	0.88	1	0.93	268	1	0.72	2	0.55	2	0.88	2	0.80	1	0.88
61	1	0.79	2	0.59	1	0.60	2	0.88	1	0.93	272	1	0.72	2	0.55	2	0.88	2	0.80	1	0.88
64	1	0.78	2	0.58	2	0.84	2	0.88	1	0.93	274	1	0.72	2	0.55	2	0.88	2	0.80	1	0.88
66	1	0.77	3	0.75	1	0.59	2	0.87	1	0.93	279	1	0.72	3	0.72	2	0.88	2	0.79	1	0.88
70	1	0.77	3	0.75	1	0.60	2	0.86	1	0.93	281	1	0.72	2	0.55	2	0.88	2	0.79	1	0.87
73	1	0.79	1	0.36	1	0.60	2	0.87	1	0.92	284	1	0.73	2	0.55	2	0.88	2	0.79	1	0.87
77	1	0.82	2	0.57	1	0.60	2	0.87	1	0.92	290	1	0.72	2	0.55	2	0.87	2	0.79	1	0.87
79	1	0.82	3	0.74	1	0.60	2	0.86	1	0.92	294	1	0.72	2	0.55	2	0.87	2	0.79	1	0.87
84	1	0.81	3	0.74	1	0.59	2	0.87	1	0.91	297	1	0.72	1	0.35	2	0.87	2	0.79	1	0.87
88	1	0.80	2	0.57	1	0.59	2	0.86	1	0.91	299	1	0.72	2	0.55	2	0.87	2	0.79	1	0.87
90	1	0.80	1	0.37	1	0.59	2	0.86	1	0.91	301	1	0.72	2	0.55	2	0.87	2	0.79	1	0.87
93	1	0.80	1	0.37	1	0.59	2	0.86	1	0.91	303	1	0.72	2	0.54	2	0.87	2	0.78	1	0.87
96	1	0.79	5	1.00	1	0.59	2	0.86	1	0.93	306	1	0.72	2	0.54	2	0.87	2	0.78	1	0.87
101	1	0.79	2	0.57	1	0.60	2	0.85	1	0.94	308	1	0.72	3	0.71	2	0.87	2	0.78	1	0.87
103	1	0.79	2	0.56	1	0.60	2	0.85	1	0.94	310	1	0.72	3	0.71	2	0.87	2	0.78	1	0.87
105	1	0.79	2	0.56	1	0.59	2	0.85	1	0.93	312	1	0.72	1	0.35	2	0.87	2	0.78	1	0.87
108	1	0.79	3	0.73	1	0.59	2	0.85	1	0.93	316	1	0.72	2	0.54	2	0.87	2	0.78	1	0.86
111	1	0.79	3	0.73	1	0.60	2	0.85	1	0.93	319	1	0.72	2	0.54	2	0.87	2	0.78	1	0.87
114	1	0.79	4	0.87	1	0.60	2	0.85	1	0.93	322	1	0.71	4	0.87	2	0.87	2	0.78	1	0.87
116	1	0.79	2	0.56	1	0.60	2	0.85	1	0.93	325	1	0.71	2	0.54	2	0.87	2	0.78	1	0.87
121	1	0.79	2	0.56	1	0.61	2	0.84	1	0.93	329	1	0.71	1	0.36	2	0.87	2	0.78	1	0.87
124	1	0.78	3	0.73	1	0.61	2	0.85	1	0.93	331	1	0.71	4	0.87	2	0.87	2	0.78	1	0.86
129	1	0.78	3	0.73	1	0.60	2	0.84	1	0.94	334	1	0.71	3	0.71	2	0.87	2	0.78	1	0.87
131	1	0.78	1	0.36	1	0.59	2	0.84	1	0.93	337	1	0.71	3	0.72	2	0.87	2	0.78	1	0.86
133	2	0.89	3	0.74	2	0.91	2	0.85	1	0.93	339	1	0.71	3	0.72	2	0.87	2	0.78	1	0.86
136	2	0.89	3	0.74	2	0.91	2	0.85	1	0.93	341	1	0.71	4	0.87	2	0.87	2	0.78	1	0.86
139	2	0.89	3	0.75	2	0.91	2	0.85	1	0.93	343	1	0.71	2	0.54	2	0.87	2	0.78	1	0.86
142	2	0.89	3	0.74	2	0.91	2	0.84	1	0.93	345	1	0.71	3	0.71	2	0.87	2	0.78	1	0.86
145	2	0.89	3	0.74	2	0.91	2	0.84	1	0.93	349	1	0.71	2	0.54	2	0.87	2	0.78	1	0.86
147	2	0.89	3	0.74	2	0.91	2	0.84	1	0.93	354	1	0.72	2	0.54	2	0.87	2	0.78	1	0.86
150	1	0.70	3	0.74	2	0.91	2	0.84	1	0.93	356	1	0.72	3	0.71	2	0.87	2	0.78	1	0.85
152	2	0.89	3	0.74	2	0.91	2	0.84	1	0.91	359	1	0.72	1	0.36	2	0.87	2	0.78	1	0.85
154	1	0.70	3	0.74	2	0.91	2	0.84	1	0.91	362	1	0.72	3	0.72	2	0.87	2	0.78	1	0.85
157	2	0.89	3	0.74	2	0.91	2	0.84	1	0.91	364	1	0.72	2	0.55	2	0.87	2	0.78	1	0.85
159	2	0.89	3	0.74	2	0.91	2	0.84	1	0.91	367	1	0.72	3	0.72	2	0.87	2	0.78	1	0.85
161	2	0.89	3	0.74	2	0.91	2	0.84	1	0.91	369	1	0.72	1	0.36	2	0.87	2	0.78	1	0.85
164	2	0.89	3	0.73	2	0.91	2	0.83	1	0.90	373	1	0.72	2	0.54	2	0.87	2	0.78	1	0.84
166	1	0.70	3	0.73	2	0.91	2	0.83	1	0.90	376	1	0.72	2	0.54	2	0.87	2	0.78	1	0.84
172	2	0.89	3	0.73	2	0.90	2	0.83	1	0.90	381	1	0.72	2	0.54	2	0.87	2	0.77	1	0.85
176	2	0.89	3	0.73	2	0.89	2	0.83	1	0.90	383	1	0.72	2	0.54	2	0.87	2	0.77	1	0.85
180	2	0.89	3	0.73	2	0.89	2	0.83	1	0.90	386	1	0.72	2	0.54	2	0.87	2	0.77	1	0.85
184	1	0.71	2	0.56	2	0.89	2	0.83	1	0.90	389	1	0.72	1	0.36	2	0.87	2	0.77	1	0.85
187	2	0.89	3	0.73	2	0.89	2	0.82	1	0.90	393	1	0.71	2	0.54	2	0.86	2	0.77	1	0.85
189	1	0.72	2	0.56	2	0.89	2	0.82	1	0.89	396	1	0.71	4	0.86	2	0.86	2	0.77	1	0.85
192	1	0.71	3	0.73	2	0.89	2	0.83	1	0.89	399	1	0.71	3	0.70	2	0.86	2	0.77	1	0.84
194	1	0.71	3	0.73	2	0.89	2	0.83	1	0.89	402	1	0.71	2	0.54	2	0.86	2	0.77	1	0.84
196	1	0.71	2	0.56	2	0.89	2	0.82	1	0.89	407	1	0.71	2	0.54	2	0.86	2	0.77	1	0.84
199	1	0.71	1	0.36	2	0.88	2	0.82	1	0.90	409	1	0.71	1	0.35	2	0.86	2	0.77	1	0.84
204	1	0.71	2	0.56	2	0.88	2	0.83	1	0.90	411	1	0.71	4	0.86	2	0.86	2	0.77	1	0.84
208	1	0.71	2	0.56	2	0.88	2	0.82	1	0.90	413	1	0.71	4	0.86	2	0.86	2	0.77	1	0.84
210	2	0.88	3	0.73	2	0.88	2	0.82	1	0.90	416	1	0.71	1	0.35	2	0.86	2	0.77	1	0.84
216	2	0.88	2	0.56	2	0.88	2	0.82	1	0.89	420	1	0.71	2	0.54	2	0.86	2	0.77	1	0.82
219	1	0.72	3	0.72	2	0.88	2	0.82	1	0.89	422	1	0.71	4	0.86	2	0.86	2	0.77	1	0.82
223	1	0.72	2	0.55	2	0.88	2	0.82	1	0.89	424	1	0.71	3	0.70	2	0.86	2	0.76	1	0.82
227	1	0.71	2	0.55	2	0.88	2	0.82	1	0.89	426	1	0.71	4	0.86	2	0.86	2	0.76	1	0.82
229	1	0.72	2	0.55	2	0.88	2	0.82	1	0.89	428	1	0.71	2	0.54	2	0.86	2	0.76	1	0.82
231	1	0.72	2	0.55	2	0.88	2	0.81	1	0.88	430	1	0.71	2	0.54	2	0.86	2	0.76	1	0.82
234	1	0.72	2	0.55	2	0.88	2	0.81	1	0.88	432	1	0.71	2	0.54	2	0.86	2	0.76	1	0.81
237	1	0.72	2	0.55	2	0.88	2	0.81	1	0.88	437	1	0.71	4	0.86	2	0.86	2	0.76	1	0.82
239	1	0.72	2	0.55	2	0.88	2	0.81	1	0.88	441	1	0.71	4	0.86	2	0.86	2	0.76	1	0.80
243	1	0.72	2	0.55	2	0.88	2	0.80	1	0.88	445	1	0.72	4	0.86	2	0.86	2	0.76	1	0.80
245	1	0.72	2	0.55	2	0.88	2	0.81	1	0.88	447	1	0.72	2	0.54	2	0.86	2	0.76	1	0.80

Table S.2. Cont.

	SES		*Et*		M_{tran}		*Mmob*		U_{case}			*SES*		*Et*		M_{tran}		*Mmob*		U_{case}	
**CL**	**n**	**s**	**n**	**s**	**n**	**s**	**n**	**s**	**n**	**s**	**CL**	**n**	**s**	**n**	**s**	**n**	**s**	**n**	**s**	**n**	**s**
449	1	0.72	2	0.53	2	0.86	2	0.76	1	0.81	653	1	0.72	2	0.53	2	0.85	2	0.74	1	0.79
453	1	0.71	1	0.35	2	0.86	2	0.76	1	0.81	656	1	0.72	2	0.53	2	0.85	2	0.74	1	0.79
455	1	0.71	2	0.53	2	0.86	2	0.76	1	0.81	659	1	0.72	2	0.53	2	0.85	2	0.73	1	0.79
457	1	0.72	2	0.53	2	0.86	2	0.76	1	0.81	662	1	0.72	4	0.86	2	0.85	2	0.74	1	0.79
461	1	0.73	2	0.53	2	0.86	2	0.76	1	0.80	664	1	0.72	2	0.53	2	0.85	2	0.74	1	0.79
466	1	0.72	4	0.86	2	0.86	2	0.75	1	0.80	669	1	0.71	2	0.53	2	0.85	2	0.73	1	0.79
471	1	0.72	2	0.53	2	0.86	2	0.75	1	0.80	672	1	0.72	2	0.53	2	0.85	2	0.73	1	0.79
474	1	0.72	4	0.86	2	0.86	2	0.75	1	0.80	674	1	0.72	2	0.53	1	0.59	2	0.73	1	0.79
477	1	0.72	2	0.53	2	0.86	2	0.75	1	0.80	676	1	0.72	3	0.70	2	0.85	2	0.73	1	0.79
480	1	0.72	2	0.53	2	0.86	2	0.75	1	0.80	679	1	0.72	4	0.86	2	0.85	2	0.73	1	0.79
484	1	0.72	2	0.54	2	0.86	2	0.75	1	0.80	681	1	0.72	1	0.35	2	0.85	2	0.73	1	0.79
488	1	0.73	2	0.53	2	0.86	2	0.74	1	0.80	688	1	0.72	2	0.53	2	0.85	2	0.73	1	0.79
494	1	0.73	1	0.35	2	0.86	2	0.75	1	0.80	690	1	0.72	3	0.70	2	0.85	2	0.73	1	0.79
497	1	0.73	1	0.35	2	0.86	2	0.74	1	0.80	693	1	0.72	2	0.53	2	0.85	2	0.73	1	0.79
500	1	0.72	2	0.53	2	0.85	2	0.74	1	0.80	695	1	0.72	2	0.53	2	0.85	2	0.73	1	0.79
506	1	0.73	4	0.86	2	0.85	2	0.74	1	0.80	698	1	0.72	2	0.53	2	0.85	2	0.73	1	0.79
509	1	0.72	4	0.86	2	0.85	2	0.74	1	0.80	701	1	0.72	2	0.53	2	0.85	2	0.73	1	0.79
511	1	0.72	2	0.53	2	0.85	2	0.74	1	0.79	709	1	0.72	4	0.86	2	0.85	2	0.73	1	0.79
516	1	0.73	4	0.86	2	0.85	2	0.75	1	0.79	711	1	0.72	2	0.53	2	0.85	2	0.73	1	0.79
520	1	0.73	4	0.86	2	0.85	2	0.75	1	0.79	714	1	0.72	2	0.53	2	0.85	2	0.73	1	0.79
523	1	0.73	4	0.86	2	0.85	2	0.75	1	0.79	718	1	0.72	2	0.53	2	0.85	2	0.73	1	0.79
525	1	0.73	4	0.86	2	0.85	2	0.75	1	0.79	722	1	0.72	2	0.53	2	0.85	2	0.73	1	0.79
528	1	0.73	2	0.53	2	0.85	2	0.75	1	0.79	725	1	0.72	2	0.53	2	0.85	2	0.73	1	0.79
531	1	0.72	1	0.35	2	0.85	2	0.74	1	0.79	728	1	0.72	2	0.53	2	0.85	2	0.73	1	0.79
533	1	0.72	3	0.70	2	0.85	2	0.74	1	0.79	731	1	0.72	2	0.53	2	0.85	2	0.73	1	0.79
536	1	0.72	1	0.35	2	0.85	2	0.74	1	0.79	735	1	0.72	2	0.53	2	0.85	2	0.73	1	0.79
541	1	0.72	4	0.86	2	0.85	2	0.74	1	0.79	737	1	0.72	2	0.53	2	0.85	2	0.73	1	0.79
544	1	0.72	3	0.70	2	0.85	2	0.74	1	0.79	746	1	0.73	2	0.53	2	0.85	2	0.73	1	0.79
546	1	0.73	1	0.35	2	0.85	2	0.74	1	0.80	749	1	0.73	4	0.86	2	0.85	2	0.73	1	0.79
550	1	0.72	4	0.86	2	0.85	2	0.74	1	0.79	753	1	0.73	4	0.86	2	0.85	2	0.73	1	0.79
554	1	0.72	3	0.70	2	0.85	2	0.74	1	0.79	756	1	0.73	4	0.86	2	0.85	2	0.73	1	0.79
556	1	0.72	4	0.86	2	0.85	2	0.74	1	0.79	760	1	0.73	4	0.86	2	0.84	2	0.73	1	0.80
558	1	0.73	2	0.53	2	0.85	2	0.74	1	0.79	763	1	0.73	2	0.53	2	0.84	2	0.73	1	0.80
560	1	0.73	1	0.35	2	0.85	2	0.74	1	0.79	766	1	0.73	1	0.35	2	0.84	2	0.73	1	0.80
562	1	0.73	3	0.70	2	0.85	2	0.74	1	0.79	770	1	0.73	1	0.35	2	0.84	2	0.73	1	0.80
564	1	0.73	1	0.35	2	0.85	2	0.74	1	0.79	775	1	0.73	2	0.53	2	0.84	2	0.73	1	0.80
566	1	0.73	3	0.70	2	0.85	2	0.74	1	0.79	782	1	0.73	4	0.86	2	0.84	2	0.73	1	0.80
568	1	0.73	1	0.35	2	0.85	2	0.74	1	0.79	785	1	0.73	2	0.53	2	0.84	2	0.73	1	0.80
570	1	0.73	3	0.70	2	0.85	2	0.74	1	0.79	789	1	0.73	2	0.53	2	0.84	2	0.73	1	0.80
572	1	0.73	1	0.35	2	0.85	2	0.74	1	0.79	792	1	0.73	4	0.86	2	0.84	2	0.73	1	0.80
575	1	0.73	2	0.53	2	0.85	2	0.74	1	0.79	796	1	0.73	3	0.70	2	0.84	2	0.73	1	0.80
578	1	0.73	2	0.53	2	0.85	2	0.74	1	0.79	798	1	0.73	3	0.70	2	0.84	2	0.73	1	0.80
581	1	0.73	2	0.53	2	0.85	2	0.74	1	0.79	803	1	0.73	2	0.53	2	0.84	2	0.73	1	0.80
586	1	0.73	4	0.86	2	0.85	2	0.74	1	0.79	805	1	0.73	2	0.53	2	0.84	2	0.73	1	0.80
588	1	0.73	2	0.53	2	0.85	2	0.74	1	0.79	807	1	0.73	4	0.86	2	0.84	2	0.73	1	0.80
591	1	0.73	2	0.53	2	0.85	2	0.74	1	0.79	810	1	0.74	3	0.70	2	0.84	2	0.73	1	0.80
593	1	0.73	4	0.86	2	0.85	2	0.74	1	0.79	812	1	0.74	3	0.70	2	0.84	2	0.73	1	0.80
596	1	0.72	4	0.86	2	0.85	2	0.74	1	0.79	821	1	0.74	3	0.70	2	0.84	2	0.73	1	0.80
598	1	0.72	2	0.53	2	0.85	2	0.74	1	0.79	824	1	0.74	4	0.86	2	0.84	2	0.73	1	0.80
601	1	0.72	2	0.54	2	0.85	2	0.74	1	0.79	827	1	0.74	4	0.86	2	0.84	2	0.73	1	0.80
607	1	0.72	4	0.86	2	0.85	2	0.74	1	0.79	830	1	0.74	4	0.86	2	0.84	2	0.73	1	0.80
610	1	0.72	4	0.86	2	0.85	2	0.74	1	0.79	837	1	0.74	2	0.53	2	0.84	2	0.73	1	0.80
612	1	0.72	2	0.54	2	0.85	2	0.74	1	0.78	843	1	0.74	4	0.86	2	0.85	2	0.73	1	0.80
615	1	0.72	2	0.54	2	0.85	2	0.74	1	0.78	849	1	0.74	2	0.53	2	0.85	2	0.73	1	0.80
619	1	0.72	4	0.86	2	0.85	2	0.74	1	0.78	855	1	0.74	4	0.86	2	0.85	2	0.73	1	0.81
621	1	0.72	1	0.36	2	0.85	2	0.74	1	0.78	858	1	0.74	3	0.70	2	0.85	2	0.73	1	0.81
623	1	0.72	2	0.54	2	0.85	2	0.74	1	0.78	860	1	0.74	4	0.86	2	0.85	2	0.73	1	0.81
625	1	0.72	2	0.54	2	0.85	2	0.74	1	0.78	868	1	0.74	2	0.53	2	0.85	2	0.73	1	0.81
634	1	0.71	4	0.86	2	0.85	2	0.74	1	0.78	876	1	0.74	3	0.70	2	0.85	2	0.73	1	0.81
637	1	0.71	2	0.53	2	0.85	2	0.74	1	0.78	881	1	0.74	3	0.70	2	0.85	2	0.73	1	0.81
641	1	0.71	2	0.53	2	0.85	2	0.74	1	0.79	886	1	0.74	4	0.86	2	0.85	2	0.73	1	0.81
645	1	0.71	4	0.86	2	0.85	2	0.74	1	0.79	888	1	0.74	3	0.70	2	0.85	2	0.73	1	0.81
647	1	0.71	4	0.86	2	0.85	2	0.74	1	0.79	890	1	0.74	3	0.70	2	0.85	2	0.73	1	0.81
651	1	0.72	2	0.53	2	0.85	2	0.74	1	0.79	895	1	0.74	3	0.70	2	0.85	2	0.73	1	0.81
