# Supplementary material for: Do More Hospital Beds Lead to Higher Hospitalization Rates? A Spatial Examination of Roemer’s Law
Source: PLoS One. 2013 Feb 13;8(2):e54900. doi: 10.1371/journal.pone.0054900 (PMC3572098; doi:10.1371/journal.pone.0054900)
Supplement: Text S1 — Enhanced Two-Step Floating Catchment Area. (PDF) [file pone.0054900.s008.pdf]

Do more hospital beds lead to higher hospitalization rates? A spatial examination of Roemer's Law

Paul L. Delamater[1,*], Joseph P. Messina[2], Sue C. Grady[1], Vince WinklerPrins[3], Ashton M. Shortridge[1]

1 Department of Geography, Michigan State University, East Lansing, Michigan, USA

2 Department of Geography, Center for Global Change and Earth Observations, Michigan AgBioResearch, Michigan State University, East Lansing, Michigan, USA

3 Georgetown University Medical Center, Washington, DC, USA

∗ **E-mail: delamate@msu.edu**

Supporting Information Text S.1

Enhanced Two-Step Floating Catchment Area

The floating catchment area (FCA) metrics are a recently-proposed set of gravity-based GIS measures of spatial accessibility. These metrics allow both availability and accessibility to be integrated by including measures of supply, demand, and distance simultaneously [1]. The general form of the gravity-based models used in their formulation can be represented as:

$$A_i^G = \sum_{j=1}^{n} \frac{S_j f(d_{i,j})}{\sum_{k=1}^{m} P_k f(d_{k,j})}, \tag{S.1}$$

where A_i^G is the spatial accessibility for population zone i, S_j is the attractiveness of a facility at location j, $f(d_{i,j})$ is an impedance (decay) function based on the distance (d) from zone i to location j, $f(d_{k,j})$ is an impedance function based on the distance from zone k to location j, and P_k is the population in zone k. The total number of zones and facilities are n and m, respectively.

One drawback in using gravity-based measures is that the output (A^G) is in units that are difficult to interpret. The FCA metrics overcome this limitation by using the general form of Eq. S.1, while producing easy to understand ratio measures. The E2SFCA improves on its predecessor, the two-step floating catchment area [2,3, 2SFCA], by integrating distance decay into the model as service area "rings" radiating from each service location. The rings are assigned weight values such that the probability of accessing a supply location is discounted with increased distance to the location.

In past research, the three functions most oftenly used to model distance decay in gravity-based measures are the Inverse power, Exponential, and Gaussian [4]. Our initial investigations showed that the oft-used distance decay functions did not adequately fit the utilization patterns of Michigan's residents. Instead, the downward log logistic function was used to model distance decay and assign weights.

After empirically estimating the parameter values of this function, we calculated weights given the travel times that were included within each unique travel ring. For example, to calculate the weight value for the 0–5 minute ring, specific weight values were calculated for each integer:

$$w_i = \frac{1}{1 + (\frac{i}{13.89})^{1.82}}, \tag{S.2}$$

where $i = \{0,1,2,3,4,5\}$ minutes travel time and:

$$W_r = \bar{w}, \tag{S.3}$$

where W_r is the weight value for the 0–5 ring and $\bar{w}$ is the mean value of the w_i values. The final set of W_r values are found in Table 1 of the main text.

The following paragraphs contain a worked example of the E2SFCA. The example illustrates the calculation of the E2SFCA for a simple system of 5 population units (each having a population of 1,000 people) and two supply locations (having 40 and 50 beds). The supply locations are located very near each other and the population units are arranged in a constellation surrounding the supply locations. Two population units fall inside the 0–5 minute travel time ring, one falls inside the 10–15 minute ring, one falls inside the 25–30 minute ring, and one falls inside the 45–60 ring of *both* hospitals.

Using the weights from Table 1 and Eq. 8, the supply ratios for each hospital are calculated:

$$R_1 = \frac{40}{1000 * 0.9459 + 1000 * 0.9459 + 1000 * 0.5511 + 1000 * 0.2253 + 1000 * 0.0832} \tag{S.4}$$

$$R_2 = \frac{50}{1000 * 0.9459 + 1000 * 0.9459 + 1000 * 0.5511 + 1000 * 0.2253 + 1000 * 0.0832}, \tag{S.5}$$

which produces ratios of $R_1 = 0.0145$ beds/person and $R_2 = 0.0182$ beds/person.

The second step of the E2SFCA calculates the availability of hospital beds for each population unit

using Eq. 9. We employ the supply ratios and the weights from the previous step:

$$A_1 = 0.0145 * 0.9459 + 0.0182 * 0.9459 \tag{S.6}$$

$$A_2 = 0.0145 * 0.9459 + 0.0182 * 0.9459 \tag{S.7}$$

$$A_3 = 0.0145 * 0.5511 + 0.0182 * 0.5511 \tag{S.8}$$

$$A_4 = 0.0145 * 0.2253 + 0.0182 * 0.2253 \tag{S.9}$$

$$A_5 = 0.0145 * 0.0832 + 0.0182 * 0.0832 \tag{S.10}$$

producing accessibility values of $A_1 = 0.03093$ beds/person, $A_2 = 0.03093$ beds/person, $A_3 = 0.01802$ beds/person, $A_4 = 0.00737$ beds/person, and $A_5 = 0.00272$ beds/person.

To test the that the E2SFCA provides an accurate and robust allocation of hospital beds to the population units, we multiply the population unit specific ratios by the population of the units. This produces 30.93, 30.93, 18.02, 7.37, and 2.72 total beds allocated for units $A_1 - A_5$ (respectively). Summing these values equals 90 total beds allocated within the system. Given that the sum of the beds in the two hospitals is also 90, this shows that the output of E2SFCA effectively distributes the resources to the population units.

References

1. Ngui A, Apparicio P (2011) Optimizing the two-step floating catchment area method for measuring spatial accessibility to medical clinics in montreal. BMC Health Services Research 11: 1–12.

2. Radke J, Mu L (2000) Spatial decompositions, modeling and mapping service regions to predict access to social programs. Annals of GIS 6: 105–112.

3. Luo W, Wang F (2003) Measures of spatial accessibility to health care in a GIS environment: synthesis and a case study in the chicago region. Environment and Planning B: Planning and Design 30: 865–884.

4. Kwan M (1998) Space-Time and integral measures of individual accessibility: A comparative analysis using a point-based framework. Geographical Analysis 30: 191–216.
